# Association of accelerometer-derived circadian abnormalities and genetic risk with incidence of atrial fibrillation

**DOI:** 10.1038/s41746-023-00781-3

**Published:** 2023-03-04

**Authors:** Lulu Yang, Hongliang Feng, Sizhi Ai, Yue Liu, Binbin Lei, Jie Chen, Xiao Tan, Christian Benedict, Ningjian Wang, Yun Kwok Wing, Lu Qi, Jihui Zhang

**Affiliations:** 1 Guangdong Cardiovascular Institute, Guangdong Provincial People’s Hospital, Guangdong Academy of Medical Sciences, Guangzhou, Guangdong China; 2 Li Chiu Kong Family Sleep Assessment Unit, Department of Psychiatry, Faculty of Medicine, The Chinese University of Hong Kong, Hong Kong SAR, China; 3 Center for Sleep and Circadian Medicine, The Affiliated Brain Hospital of Guangzhou Medical University, Guangzhou, Guangdong China; 4 Department of Cardiology, Heart Center, The First Affiliated Hospital of Xinxiang Medical University, Weihui, Henan China; 5 Department of Psychiatry, Faculty of Medicine, The Chinese University of Hong Kong, Hong Kong SAR, China; 6 Department of Big Data in Health Science, Zhejiang University School of Public Health and Department of Psychiatry, Sir Run Run Shaw Hospital, Zhejiang University School of Medicine, Hangzhou, China; 7 Department of Medical Sciences, Uppsala University, Uppsala, Sweden; 8 Molecular Neuropharmacology (Sleep Science Laboratory), Department of Pharmaceutical Biosciences, Uppsala University, Uppsala, Sweden; 9 Institute and Department of Endocrinology and Metabolism, Shanghai Ninth People’s Hospital, Shanghai JiaoTong University School of Medicine, Shanghai, China; 10 Department of Epidemiology, School of Public Health and Tropical Medicine, Tulane University, New Orleans, LA USA; 11 Department of Nutrition, Harvard T.H. Chan School of Public Health, Boston, MA USA; 12 Guangdong Mental Health Center, Guangdong Provincial People’s Hospital (Guangdong Academy of Medical Sciences), Southern Medical University, Guangzhou, Guangdong China; 13 Key Laboratory of Neurogenetics and Channelopathies of Guangdong Province and the Ministry of Education of China, Guangzhou Medical University, Guangzhou, China

**Keywords:** Risk factors, Atrial fibrillation

## Abstract

Evidence suggests potential links between circadian rhythm and atrial fibrillation (AF). However, whether circadian disruption can predict the onset of AF in the general population remains largely unknown. We aim to investigate the association of accelerometer-measured circadian rest-activity rhythm (CRAR, the most prominent circadian rhythm in humans) with the risk of AF, and examine joint associations and potential interactions of CRAR and genetic susceptibility with AF incidence. We include 62,927 white British participants of UK Biobank without AF at baseline. CRAR characteristics, including amplitude (strength), acrophase (timing of peak activity), pseudo-F (robustness), and mesor (height), are derived by applying an extended cosine model. Genetic risk is assessed with polygenic risk scores. The outcome is the incidence of AF. During a median follow-up of 6.16 years, 1920 participants developed AF. Low amplitude [hazard ratio (HR): 1.41, 95% confidence interval (CI): 1.25–1.58], delayed acrophase (HR: 1.24, 95% CI: 1.10–1.39), and low mesor (HR: 1.36, 95% CI: 1.21–1.52), but not low pseudo-F, are significantly associated with a higher risk of AF. No significant interactions between CRAR characteristics and genetic risk are observed. Joint association analyses reveal that participants with unfavourable CRAR characteristics and high genetic risk yield the highest risk of incident AF. These associations are robust after controlling for multiple testing and in a series of sensitivity analyses. Accelerometer-measured CRAR abnormalities, characterized by decreased strength and height, and later timing of peak activity of circadian rhythm, are associated with a higher risk of AF in the general population.

## Introduction

Atrial fibrillation (AF), one of the most common cardiac arrhythmias, is a major cause of morbidity, mortality, and health care expenditure^1^. Thus, the prevention of AF is an urgent public health priority. Intensive research over the previous decades has identified multiple modifiable risk factors for AF, such as smoking, excessive alcohol use, obesity, hypertension, and diabetes mellitus^2^. However, the prevalence and incidence of AF are increasing rapidly^3,4^, and therefore more efforts should be undertaken to identify novel risk factors.

In recent years there has been growing interest in the association between circadian rhythm and AF. Circadian rhythm refers to physiological and behavioural cycles with approximately 24 h related to the light-dark cycle of earth^5^. Compelling evidence demonstrates that almost all cardiovascular variables in humans exhibit circadian fluctuations, including blood pressure^6^, heart rate^6^, circulating catecholamines^7^, and vascular endothelial function^8^. Epidemiological data also revealed a prominent circadian variation in the occurrence of AF episodes^9^. In addition, animal studies have proven the important role of circadian rhythm in arrhythmia pathogenesis^10,11^. Recently, a case-control study^12^ with a small sample size observed circadian rhythm changes among patients with AF, and our team identified that night shift workers exhibited an increased AF risk, further suggesting a link between circadian rhythm and AF^13^. However, to date, whether circadian rhythm disruption can predict the onset of AF in the general population remains largely unknown, as studying circadian rhythms in humans is hampered by difficulty in collecting serial samples across different time points. Furthermore, AF is a complex disease with shared environmental and genetic factors that contribute to disease pathogenesis^14^. However, it remains unknown whether genetic risk may modify the effect of circadian rhythm on AF risk.

The UK Biobank has collected 7-day activity data from over 100,000 participants with wrist-worn accelerometers that can continuously measure human rest and activity cycles with a good estimate of the circadian rest-activity rhythm (CRAR, the most prominent circadian rhythm in humans)^15^. In addition, in-depth genetic data are available for UK Biobank participants. Therefore, the UK Biobank provides an unprecedented opportunity to test the association of disrupted circadian rhythm and genetic risk with AF. Based on the UK Biobank study, we aim: 1) to investigate the association of a series of CRAR parameters, including amplitude (strength), acrophase (timing of peak activity), pseudo-F (robustness), and mesor (height), with incident AF; and 2) to assess their joint associations and potential interactions between CRAR and genetic susceptibility with the risk of AF. Our results demonstrate that CRAR abnormalities, hallmarked by low amplitude, delayed acrophase, and low mesor, but not pseudo-F, were strongly associated with AF risk. No significant interactions between CRAR characteristics and genetic risk are observed. Joint association analyses reveal that participants with unfavourable CRAR characteristics and high genetic risk yield the highest risk of incident AF.

## Results

### Population Characteristics

From the 92,614 participants with valid data on circadian rest-activity parameters, 27,953 participants were excluded because of failure in genetic quality control, and 1734 were excluded because they were diagnosed with AF before wearing the accelerometer (Supplementary Fig. 1), leaving 62,927 participants for the main analyses. The mean (standard deviation, SD) age was 62.48 (7.75) years, and 35,323 (56.13%) participants were female. During a median (interquartile range) follow-up of 6.16 (5.60–6.68) years, 1920 participants (3.05%) developed AF.

Baseline characteristics of the study population according to incident AF are provided in Table 1. Compared with participants without incident AF, those with incident AF were more likely to be older, male, materially deprived, and English. They also tended to be more obese, smokers, have lower education levels, have a less healthy diet, consume more coffee and tea, and were more likely to have hypertension, diabetes, and dyslipidaemia. Regarding the alcohol consumption, participants with incident AF more rarely consumed alcohol two or fewer times per week but more often reported drinking alcohol at least three times per week compared to those without incident AF. In addition, participants with incident AF appeared to have lower sleep efficiency and abnormal sleep duration (<7 h/day or >8 h/day).

**Table 1:** Characteristics of participants at baseline.

Characteristic	No. (%)		
	All (*n* = 62,927)	Participants without AF (*N* = 61,007)	Participants with AF (*n* = 1920)
Age at accelerometer, mean (SD), y	62.48 (7.75)	62.31 (7.73)	67.90 (5.94)
Female	35,323 (56.13)	34,594 (56.70)	729 (37.97)
Townsend deprivation index^a^, mean (SD)	−1.91 (2.71)	−1.91 (2.71)	−1.86 (2.69)
Recruitment regions			
England	56,207 (89.32)	54,408 (89.18)	1799 (93.70)
Wales	2489 (3.96)	2469 (4.05)	20 (1.04)
Scotland	4231 (6.72)	4130 (6.77)	101 (5.26)
Education level			
Degree or above	26,998 (42.90)	26,280 (43.08)	718 (37.40)
Any other qualification	30,723 (48.82)	29,774 (48.80)	949 (49.43)
No qualification	5206 (8.27)	4953 (8.12)	253 (13.18)
Season of accelerometer wear			
Spring	14,316 (22.75)	13,899 (22.78)	417 (21.72)
Summer	16,430 (26.11)	15,906 (26.07)	524 (27.29)
Autumn	18,737 (29.78)	18,147 (29.75)	590 (30.73)
Winter	13,444 (21.36)	13,055 (21.40)	389 (20.26)
Body mass index^b^ categories			
Normal/Underweight (<25 kg/m^2^)	25,072 (39.84)	24,509 (40.17)	563 (29.32)
Overweight (25–30 kg/m^2^)	25,863 (41.10)	52,067 (41.09)	796 (41.46)
Obese (≥30 kg/m^2^)	11,992 (19.06)	11,431 (18.74)	561 (29.22)
Healthy diet score, mean (SD)	2.67 (1.17)	2.67 (1.17)	2.66 (1.17)
Smoking status			
Never	36,530 (58.05)	35,598 (58.35)	932 (48.54)
Previous	22,515 (35.78)	21,647 (35.48)	868 (45.21)
Current	3882 (6.17)	3762 (6.17)	120 (6.25)
Alcohol consumption			
Not current	3333 (5.30)	3223 (5.28)	110 (5.73)
Two or less times a week	28,621 (45.48)	27,812 (45.59)	809 (42.14)
Three or more times a week	30,973 (49.22)	29,972 (49.13)	1001 (52.14)
Coffee consumption (Yes)	51,339 (81.59)	49,753 (81.55)	1586 (82.60)
Tea consumption (Yes)	53,906 (85.67)	52,250 (85.65)	1656 (86.25)
Hypertension history	16,750 (26.62)	15,886 (26.04)	864 (45.00)
Diabetes history	2833 (4.50)	2650 (4.34)	183 (9.53)
Dyslipidaemia history	8504 (13.51)	8055 (13.20)	449 (23.39)
Sleep efficiency, mean (SD)	0.76 (0.07)	0.76 (0.07)	0.75 (0.08)
Sleep duration			
< 7 h/day	21,076 (33.49)	20,354 (33.36)	722 (37.60)
7–8 h/day	29,075 (46.20)	28,272 (46.34)	803 (41.82)
> 8 h/day	12,776 (20.30)	12,381 (20.29)	395 (20.57)

^a^Townsend deprivation index was calculated based on the preceding national census output areas prior to participants joining UK Biobank.

^b^Body mass index is calculated as weight in kilograms divided by height in meters squared.

### Associations of genetic risk with incident atrial fibrillation

As shown in Table 2, AF risk increased monotonically across genetic risk categories. Participants with a high genetic risk had a 2.52-fold increase in AF risk compared with those with a low genetic risk (HR: 2.52, 95% CI: 2.25–2.83). Additional adjustment for circadian rest-activity parameters did not change these results, indicating that genetic risk for AF was statistically independent of CRAR.

**Table 2:** Risk of incident atrial fibrillation according to genetic risk.

Genetic risk	No. of AF cases/ person-years	Model 1 HR (95% CI); *P*	Model 2 HR (95% CI); *P*	Model 3 HR (95% CI); *P*	Model 4 HR (95% CI); *P*
Low genetic risk (*N* = 21,274)	410/129,366	1[Reference]	1[Reference]	1[Reference]	1[Reference]
Intermediate genetic risk (*N* = 21,091)	561/127,971	1.41 (1.24, 1.60); <0.001	1.41 (1.24, 1.60); <0.001	1.40 (1.24, 1.59); <0.001	1.40 (1.23, 1.59); <0.001
High genetic risk (*N* = 20,562)	949/123,673	2.52 (2.25, 2.83); <0.001	2.52 (2.25, 2.83); <0.001	2.54 (2.26, 2.85); <0.001	2.54 (2.26, 2.85); <0.001
*P* value for trend		<0.001	<0.001	<0.001	<0.001

Analyses were conducted using Cox proportional hazard models. Model 1 was adjusted for age, sex, and first 10 principal components of ancestry. Model 2 was adjusted as in model 1 and for Townsend deprivation index, recruitment centre, education level, and season of accelerometer wear. Model 3 was adjusted as in model 2 and for BMI categories, healthy diet score, smoking status, alcohol intake, coffee consumption, tea consumption, hypertension, diabetes, and dyslipidemia. Model 4 was adjusted as in model 3 and for sleep efficiency, sleep duration, amplitude, acrophase, F-Pseudo, and mesor. Participants with prevalent AF were excluded.

*AF* atrial fibrillation, *BMI* body mass index, *CI* confidence interval, *HR* hazard ratio.

### Associations of circadian rest-activity rhythm with incident atrial fibrillation

**Fig. 1:**
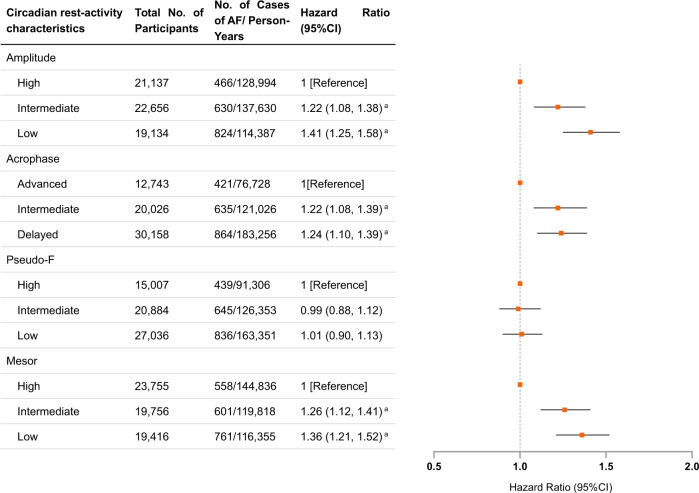
Risk of incident atrial fibrillation according to circadian rest-activity characteristics. AF atrial fibrillation, BMI body mass index, CI confidence interval, FDR false-discovery rate, HR hazard ratio. ^a^*P* values remained significant after multiple testing with FDR method. Data are presented as HRs and 95% CI. Cox proportional hazards models were adjusted for age, sex, Townsend deprivation index, risk, recruitment centre, education level, season of accelerometer wear, BMI categories, healthy diet score, smoking status, alcohol intake, coffee consumption, tea consumption, hypertension, diabetes, dyslipidemia, sleep efficiency, sleep duration, genetic risk, and first 10 principal components of ancestry for AF. Participants with prevalent AF were excluded.

Supplementary Fig. 2 shows the acceleration level during 7-day monitoring, with fitted curves for CRAR parameters for participants without and with incident AF. As shown in Fig. 1 and Supplementary Table 1, participants with low amplitude exhibited a higher risk of AF than those with high amplitude (HR: 1.41, 95% CI: 1.25–1.58). Similarly, delayed acrophase and low mesor were also strongly associated with a higher risk of AF (delayed vs. advanced acrophase: HR: 1.24, 95% CI: 1.10–1.39; low vs. high mesor: HR: 1.36, 95% CI: 1.21–1.52). However, no significant association between pseudo-F and the risk of AF was observed. These associations remained consistent even after false-discovery rate (FDR) correction. Those results with additional adjustment for genetic risk demonstrated that the associations of amplitude, acrophase, and mesor with the risk of AF were statistically independent of genetic risk. In addition, subgroup analyses stratified by age and sex categories revealed similar results, indicating that the findings were consistent across age and sex (Supplementary Tables 2 and 3).

### Joint association and interaction of genetic risk and circadian rest-activity rhythm with incident atrial fibrillation

**Fig. 2:**
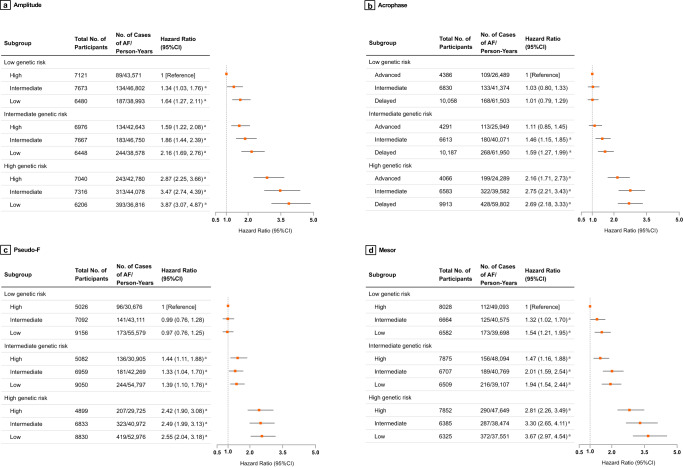
Risk of incident atrial fibrillation according to genetic and circadian rest-activity characteristics. AF atrial fibrillation, BMI body mass index, CI confidence interval, FDR false-discovery rate, HR hazard ratio. ^a^*P* values remained significant after multiple testing with FDR method. Data are presented as HRs and 95% CI. The joint associations of amplitude (**a**), acrophase (**b**), Pseudo-F (**c**), and mesor (**d**) as well as genetic risk with incidence of AF among 62,927 participants from UK Biobank. Cox proportional hazards models were adjusted for age, sex, Townsend deprivation index, recruitment centre, education level, season of accelerometer wear, BMI categories, healthy diet score, smoking status, alcohol intake, coffee consumption, tea consumption, hypertension, diabetes, dyslipidemia, sleep efficiency, sleep duration, and first 10 principal components of ancestry. Participants with prevalent AF were excluded.

Figure 2 and Supplementary Table 4 show the risk of incident AF for the combined genetic risk and CRAR categories. For amplitude, the highest risk of incident AF was observed among participants with high genetic risk and low amplitude (HR: 3.87, 95% CI: 3.07–4.87). Participants with high genetic risk and delayed acrophase had a 2.69-fold higher risk of AF than those with low genetic risk and advanced acrophase (HR: 2.69, 95% CI: 2.18–3.33). Participants with high genetic risk and low pseudo-F exhibited a 2.55-fold increase in AF risk compared to participants with low genetic risk and high pseudo-F (HR: 2.55, 95% CI: 2.04–3.18). Participants with high genetic risk and low mesor had a 3.67-fold higher risk of incident AF compared to participants with low genetic risk and high mesor (HR: 3.67, 95% CI: 2.97–4.54). These associations were statistically significant upon FDR correction. Cumulative incidence curves of incident AF according to genetic risk and CRAR categories are shown in Supplementary Figs. 3–6. All results were robust in a series of sensitivity analyses by using competing risk regression or restricted to participants without any missing covariate data, or by excluding events that occurred within the first year of follow-up, or by censoring up to December 31, 2019, or by excluding those with a self-report history of shift work (Supplementary Tables 5–9).

There was no significant interaction between CRAR and the genetic risk score (overall *P* for interaction >0.05, Cox regression analysis), indicating that the associations with CRAR did not vary substantially according to genetic risk. Further analyses stratified by genetic risk category with strong CRAR (i.e., high amplitude, advanced acrophase, high pseudo-F, and high mesor) as the reference groups suggested that low amplitude, delayed acrophase, and low mesor were associated with a higher AF risk across genetic groups, especially intermediate and high genetic risk groups. However, no significant associations were detected between pseudo-F and the risk of AF among different genetic groups (Supplementary Table 10).

## Discussion

In this large population-based cohort, we found that CRAR abnormalities, hallmarked by low amplitude, delayed acrophase, and low mesor (but not pseudo-F), were strongly associated with AF risk. When examining the joint associations of CRAR and genetic risk with AF incidence, high genetic risk in combination with CRAR abnormalities was associated with a more than 2.5-fold greater risk of AF compared with low genetic risk and normal CRAR. These associations were rather consistent and robust even after controlling for multiple testing and in a series of sensitivity analyses. There was no evidence of interactions between CRAR and genetic risk, and the CRAR abnormalities were associated with a higher risk of AF regardless of genetic risk.

Our findings add to a growing body of literature suggesting a close link between disrupted circadian rhythm and AF. For instance, Chen et al.^12^ found decreased expression of circadian clock genes among patients with AF and observed a significant correlation between the decreased expression of circadian clock genes and higher atrial high-rate episodes. Furthermore, recent research among nurses suggested that shift work was associated with similar circadian abnormalities, including low amplitude, delayed phase, and low mesor^16^. In this regard, night shift workers, had an elevated risk of AF, regardless of genetic AF risk^13^, further echoing the findings of the current study. More importantly, our study further extends the published reports by providing evidence of the strong associations of the objectively measured circadian rhythm abnormalities with AF onset regardless of genetic risk, highlighting the important role of circadian rhythm in the development of AF in the general population.

Our findings are also supported by several previous studies that assessed the relationship of physical activity and sleep with AF^17–19^, as physical activity and sleep are known to be regulated by circadian rhythm^20^. Specifically, Khurshid et al.^17^ found that physical activity, which is linked to the strength and height of CRAR, was strongly associated with a lower risk of AF. Furthermore, by using continuous monitoring for AF episodes and accelerometer-measured physical activity for approximately 3.5 years, Bonnesen et al. found that a within-individual decrease in daily physical activity can also predict the onset of AF episodes among high-risk populations^18^. In addition, Li et al. found that early chronotype, was associated with a decrease in AF risk, which is in line with our findings of the association between acrophase and AF onset^19,21^. Our findings suggest that the role of circadian disruption in the development of AF adds value to traditional sleep and physical activity indices.

The precise mechanisms by which circadian rhythm abnormalities increase the risk for AF remain unclear. Accumulating studies have identified a direct role of the biological clock in regulating cardiac metabolism, growth, and response to injury^22^. Notably, evidence also suggests that circadian rhythm regulates the activity of the autonomic nervous system and the various cardiac ion channels, which are associated with the pathophysiologic mechanism of AF^10,22,23^. In addition, a broad range of metabolic changes, such as cortisol, vascular inflammation, and oxidative stress, may explain the observed link between circadian disruption and AF^24–26^. Nonetheless, more evidence is warranted to identify a complete profile of circadian rhythm disruption and genetic risk in the development of AF.

This is a large prospective analysis to examine the combined role of objectively measured circadian rhythm abnormalities and genetic risk on the risk of AF in the general population. Our study has several public health implications for AF prevention. First, the current study found that accelerometer-measured circadian abnormalities predicted the occurrence of AF. Rapid advances in documenting circadian rest-activity patterns by mobile technology such as accelerometers, may therefore provide valuable screening tools to identify at-risk individuals. Second, the results of the current study, together with prior evidence, emphasize the importance of improving circadian function in the prevention of AF, and lend support to potential interventions targeting the improvement of circadian rhythm in the prevention of AF risk in the general population.

The major strengths of this study include the large sample size, the prospective and population-based study design, and a careful consideration of potential confounding factors. In addition, the circadian rhythm as the exposure was objectively measured using accelerometers. Our study also has several limitations. First, the UK Biobank is not representative of the population in other respects with evidence of a ‘selection’ or ‘healthy volunteer’ bias^27,28^. However, the valid estimates and comparisons of exposure-disease relationships are widely generalizable in this sample^29^. Second, some covariates such as lifestyle were not collected at the present study baseline (accelerometer mail-out) but the physical visits to the UK Biobank assessment centres, a median of 5.7 years prior. Nonetheless, the responses were generally stable over time^30^. In addition, despite including a wide range of confounders in the analyses, residual or unmeasured confounding could not be ruled out in our study, such as sleep medication (e.g., benzodiazepines). Third, as with any observational study, causal inference cannot be made in our study. Despite this, we attempted to minimize this risk by adjusting for potential confounding factors and the results also remained unchanged when we excluded participants with outcome events that occurred during the first year of follow-up. Fourth, incident AF cases were ascertained through hospital inpatient records, surgical records, and death registry only and some cases of AF were likely to have been missed. Nevertheless, the AF rates we observed in our sample were generally consistent with population-based estimates within similar age groups^31,32^, suggesting that the selection bias was minimal. Furthermore, misclassification errors were likely to have biased these findings towards the null and would underestimate the risk associated with the CRAR profile. Fifth, although an accelerometer allows us to continuously measure circadian rhythm of rest-activity in the free-living environment among a scalable population, it should be noted that CRAR is susceptible to masking effects from imposed social schedules, and so it may be less stable than other typical measures of rhythms, such as dim light melatonin secretion and core body temperature. It is important to study the role of circadian rhythm in the onset of AF by further investigation with more reliable circadian markers. Sixth, the duration of accelerometer monitoring was limited, and it is still unclear whether a seven-day measurement is representative of long-term behaviour, especially CRAR parameters, although previous evidence demonstrates that a seven-day monitoring period has been routinely used in activity monitoring studies and provides a sufficiently large number of days to achieve a high level of intra-class correlations in most populations^33^. Further investigation with repeated or long-term accelerometer monitoring is of great importance in understanding the associations between CRAR and AF. Finally, as those AF-related SNPs were selected from individuals of European descent, the generalizability of the study findings to other populations should be evaluated in future studies.

In sum, we found that accelerometer-measured CRAR abnormalities, characterized by decreased strength and height, and later timing of peak activity of circadian rhythm, were significantly associated with the future risk of AF in the general population. Our findings highlight the potential of circadian intervention to reduce the risk of AF.

## Methods

### Study population

The UK Biobank is a population-based prospective study with over 500,000 participants aged 40–73 years recruited in 2006–2010^34^. Participants underwent detailed baseline assessments including various sociodemographic, lifestyle, health, and physical assessments through touch-screen questionnaires and physical measurements. Further details of the study are available online (www.ukbiobank.ac.uk). Between February 2013 and December 2015 (on average, approximately 5.5 years after their baseline recruitment), 236,519 UK Biobank participants were invited to participate in an accelerometer study. A total of 106,053 (44.8%) participants agreed to take part and were provided with a wrist-worn Axivity AX3 accelerometer (Axivity, Newcastle upon Tyne, UK). Finally, 103,712 raw accelerometer datasets were received for data analysis^35^. Participants who accepted to undergo accelerometer measurement showed similar baseline demographic and health-related characteristics as those who declined the measurement^17^.

Using the raw accelerometer data, the UK Biobank accelerometer expert working group conducted data processing and generated the physical activity intensity data in 5-s epochs for 103,682 participants. The flowchart of participant selection of the current study is shown in Supplementary Fig. 1. Based on the data quality metrics provided by the UK Biobank accelerometer working group, the exclusion criteria were as follows: 1) those data flagged by the UK Biobank as being unreliable due to unexpectedly small or large size; 2) those with accelerometer data for less than 72 h or did not provide data for all 1-h periods within a 24-h cycle during the 7-day data collection; 3) those data identified by the UK Biobank as not being well-calibrated; 4) those data were recalibrated using the previous accelerometer record from the same device worn by a different participant; 5) those data with a non-zero count of interrupted recording periods; and 6) those data with more than 768 (Q3 + 1.5 × IQR) data recording errors. Furthermore, during the quality control process of genetic data, participants with missingness (>10%), outliers for heterozygosity, biologically related, and those whose reported sex was inconsistent with sex inferred from the genetic data as well as those with sex chromosome aneuploidy, those who were genetically defined as not white British, and those with prevalent AF based on self-report or medical records were also excluded. Finally, 62,927 participants were included in our study.

The UK Biobank received ethical approval from the NHS (National Health Service) National Research Ethics Service (Ref11/NW/0382). All participants gave written informed consent before enrolment in the study, which was conducted in accordance with the principles of the Declaration of Helsinki. This study was performed under UK Biobank application number 58082.

### Circadian rest-activity rhythm

CRAR characteristics were derived by applying an extended cosine model analysis to the activity data, which has been used extensively in prior studies^36,37^. This method applies an antilogistic transformation to the cosine curve and allows for greater flexibility in fitting the data, and therefore is more suitable for studying rhythms in the older population, whose diurnal patterns tend to deviate from a cosine shape^38^. We focused on four key parameters: 1) amplitude, a measure of the strength of the rhythm, is the peak-to-nadir difference in activity of the fitted curve, and higher values indicated larger rhythm strength with a higher activity level during the day and a lower activity level during the night; 2) acrophase, the timing of peak activity of the fitted curve, was measured in portions of hours (time of day), and earlier or later times may reflect an advanced or delayed peak of rhythm respectively; 3) pseudo-F, a model goodness-of-fit measure and an indicator of overall rhythmicity, and higher pseudo-F values indicate more regular activity patterns that can be modelled by a function with a 24-h period, indicating more robust rhythms; and 4) mesor (midline estimating statistic of rhythm), the mean level of activity of the fitted 24-hour rest-activity pattern and reflecting the central tendency of an oscillating variable, represents the height of the rhythm. Higher values indicate more robust activity levels. The data were analysed using the ‘ActCR’ R package^39^.

### Genotyping data

Genetic data on 488,377 UK Biobank participants were generated using two genotyping arrays. The Affymetrix UK BiLEVE Axiom Array returned genotypes at 807,411 markers on 49,950 participants^40^. The Affymetrix UK Biobank Axiom Array provided genotypes at 825,925 markers for the remaining 438,427 participants. Because these platforms shared 95% of genetic markers, quality controls and imputation (the determination of genotypes at loci by inference and not by direct genotyping) were performed jointly, which have been previously described in detail^41^. Specifically, imputed genotype data were provided by the UK Biobank, based on merged UK10K and 1000 Genomes phase 3 panels.

### Genetic risk score

The weighted genetic risk score (GRS) was created for AF using single-nucleotide polymorphisms (SNPs) associated with AF at the genome-wide association significance in a meta-analysis of genome-wide association studies of individuals of European ancestry that did not include participants from the UK Biobank^42^. Therefore, the current study was restricted to participants who were genetically defined as white British. Information on the 166 selected SNPs is listed in Supplementary Table 11. Individual SNPs were coded as 0, 1, and 2 according to the number of risk alleles. Missing allele dosages were imputed and replaced with the mean value across the respective SNPs. The regression coefficient for each SNP was taken from the reported meta-analysis^42^. The number of associated alleles at each SNP was weighted according to the estimated effect size in the reported meta-analysis^42^. This genetic risk score was then categorized into “low risk” (lowest third), “intermediate risk” (second third), and “high risk” (highest third).

### Incident atrial fibrillation

The outcome was the diagnosis of AF, which was defined as either AF or atrial flutter^17^. Incident AF in the UK Biobank was ascertained through hospital inpatient and death records. In detail, incident AF was defined as International Classification of Diseases (ICD) edition 10 (codes I48, I48.0, I48.1, I48.2, I48.3, I48.4, I48.9) and operative procedures (codes K62.1, K62.2, K62.3, K62.4). More details are shown in Supplementary Table 12. Particularly, ICD 9 and self-report diagnoses were used only to ascertain the presence of AF at baseline for exclusion from incident analyses. At the time of analysis, hospital admission data were available for participants until 15 January 2021. Therefore, participants were censored at the date associated with the development of AF, date of death, or last known follow-up (15 January 2021), whichever occurred first.

### Ascertainment of covariates

We used the self-report questionnaires, accelerometer-measured variables, and medical history to assess possible confounders. Age (Continuous) was calculated from dates of birth and wearing an accelerometer. Sex (Female/Male), Townsend deprivation index reflecting socioeconomic status (Continuous), region of the UK biobank assessment centre (England/Wales/Scotland), educational attainment (Degree or above/Any other qualification/No qualification), body-mass index (BMI) categories [Normal/Underweight (<25 kg/m^2^)/ Overweight (25≤ to <30 kg/m^2^)/ Obese (≥30 kg/m^2^)], smoking status (Never/Previous/Current), frequency of alcohol intake (Not current/Less than three times a week/Three or more times a week), coffee consumption (Yes/No), tea consumption (Yes/No), and diet-related factors were obtained from touchscreen questions at the time-point closest to the accelerometer (Supplementary Fig. 7). The healthy diet score was calculated by using the following factors: vegetable intake at least four tablespoons each day (Median); fruits intake at least three pieces each day (Median); fish intake at least twice each week (Median); unprocessed red meat intake no more than twice each week (Median); and processed meat intake no more than twice each week (Median). One point was given for each favourable diet factor, with the total diet score ranging from 0 to 5. Health-related variables including hypertension, diabetes, and dyslipidaemia were obtained from the self-report questionnaires, interviews, and hospital records that were diagnosed before the accelerometer. Season of accelerometer wear (Spring/Summer/Autumn/Winter), sleep efficiency (Continuous), sleep duration (<7 h/day, 7–8 h/day, >8 h/day) recorded by the accelerometer, and the first 10 principal components of ancestry were also included as confounders.

### Statistical analyses

Baseline characteristics are presented as the mean ± SD for continuous variables and the number (%) for categorical variables. To minimize the potential for inferential bias and to maximize the statistical power possible, we conducted multiple imputations to assign any missing covariate values by using the “mice” R package^43^. Detailed information on missing covariates is shown in Supplementary Table 13. As previously described^44,45^, to account for possible non-linear associations and to ensure robustness against outliers, we trichotomized circadian rest-activity parameters using several cut-offs, which were based on change points at the associations with AF (Supplementary Fig. 8): amplitude (≤35, 35< to ≤50, >50, counts/min), acrophase (≤13:00, 13:00< to ≤14:00, >14:00, hh:mm), pseudo-F (≤100, 100< to ≤200, >200), and mesor (≤23, 23< to ≤30, >30, counts/min). If no relevant change points were found, we selected cut-offs to balance the sample size between groups.

Cox proportional hazards regression, with time since accelerometer wearing time as the start of follow-up, was used to model the associations of CRAR categories, genetic risk categories, and their combination with incident AF. Participants with AF at baseline were excluded. Moreover, we included an interaction term in the regression models to test for statistical interaction between CRAR and genetic risk scores, and we also further conducted the main analyses stratified by genetic risk category. The proportionality of hazards assumption was assessed using the Schoenfeld residuals technique^46^ and no violation of the assumption was found. Hazard ratios (HRs) and corresponding 95% confidence intervals (95% CIs) were calculated. Cox regression analyses were adjusted for age, sex, Townsend deprivation index, recruitment centre, education level, season of accelerometer wear, BMI category, healthy diet score, smoking status, alcohol intake, coffee consumption, tea consumption, hypertension, diabetes, dyslipidaemia, sleep efficiency, sleep duration, and the first 10 principal components of ancestry. Collinearity between all the covariates included in the analyses was examined via correlation matrix analysis, which revealed no problem of multicollinearity. To account for multiple testing, *P* values were corrected via the FDR by using the Benjamini–Hochberg method^47^. Based on the final model, adjusted cumulative incidence curves were then generated to show the standardized risk of incident AF according to genetic risk and different CRAR categories.

Furthermore, five sensitivity analyses were performed to investigate potential sources of bias in our results. First, to interrogate the potential bias from the competing risks, Fine-Gray subdistribution hazards were calculated, incorporating death as a competing risk for the incidence of AF. Second, we restricted the analyses to participants without any missing covariate data. Third, we excluded events that occurred within one year after accelerometer assessment to minimize the risk of reverse causality. Fourth, we performed sensitivity analyses by censoring up to December 31, 2019 (we considered this date the start of the COVID-19 epidemic) to minimize the possible bias caused by the pandemic. Fifth, we excluded participants who reported a history of shift work and repeated the main analyses.

To test the robustness and potential variations in different subgroups, we repeated the main analyses stratified by age (< 65/≥ 65 years) and sex (Female/Male). We conducted all statistical analyses by using R software version 4.0.4 and SPSS 26.0. All statistical tests were two-sided, and a *P* value of less than 0.05 was regarded as statistically significant.

### Reporting summary

Further information on research design is available in the Nature Research Reporting Summary linked to this article.

## Supplementary information

Supplementary Material

REPORTING SUMMARY

## Data Availability

This research has been conducted using the public UK Biobank Resource under Application Number 58082. Individual-level data from the UK Biobank are not publicly available according to their policy, but the data will be made available after the application of UK Biobank. Please see the website for application procedures (www.ukbiobank.ac.uk).

## Code availability

All the code for the analyses in this study are available from the authors upon the request.
