# Supplementary material for: Association of accelerometer-derived circadian abnormalities and genetic risk with incidence of atrial fibrillation
Source: NPJ Digit Med. 2023 Mar 4;6:31. doi: 10.1038/s41746-023-00781-3 (PMC9984286; doi:10.1038/s41746-023-00781-3)
Supplement: Supplementary file 1 — Supplementary Material [file 41746_2023_781_MOESM1_ESM.pdf]

Supplementary Online Content

Supplementary Figure 1. Flowchart for the selection of the analyzed study sample from the UK Biobank Study
Supplementary Figure 2. Acceleration level during seven-day monitoring with fitted curve for the circadian rest-activity characteristics among participants without and with incident atrial fibrillation
Supplementary Figure 3. Standardized risk of incident atrial fibrillation according to genetic risk and amplitude
Supplementary Figure 4. Standardized risk of incident atrial fibrillation according to genetic risk and acrophase
Supplementary Figure 5. Standardized risk of incident atrial fibrillation according to genetic risk and pseudo-F
Supplementary Figure 6. Standardized risk of incident atrial fibrillation according to genetic risk and mesor
Supplementary Figure 7. Timeline of covariates collection
Supplementary Figure 8. Associations between circadian rest-activity characteristics and incident atrial fibrillation
Supplementary Table 1. Risk of Incident atrial fibrillation according to circadian rest-activity characteristics
Supplementary Table 2. Risk of Incident atrial fibrillation according to circadian rest-activity characteristics stratified by age categories
Supplementary Table 3. Risk of Incident atrial fibrillation according to circadian rest-activity characteristics stratified by sex categories
Supplementary Table 4. Risk of Incident atrial fibrillation according to genetic and circadian rest-activity characteristics
Supplementary Table 5. Sensitivity analysis on the association of circadian rest-activity characteristics and genetic risk with Incident atrial fibrillation by using competing risk regression (Fine and Gray)
Supplementary Table 6. Sensitivity analysis on the associations of circadian rest-activity characteristics and genetic risk with incident atrial fibrillation among participants without any missing covariate data
Supplementary Table 7. Sensitivity analysis on the associations of circadian rest-activity characteristics and genetic risk with incident atrial fibrillation by excluding events that occurred within the first year of follow-up
Supplementary Table 8. Sensitivity analysis on the associations of circadian rest-activity characteristics and genetic risk with atrial fibrillation by censoring up to Dec 31, 2019
Supplementary Table 9. Sensitivity analysis on the associations of circadian rest-activity characteristics and genetic risk with atrial fibrillation by excluding those reporting a history of shift work
Supplementary Table 10. Risk of incident atrial fibrillation according to circadian rest-activity characteristics within each genetic risk category

Supplementary Table 11. Single-nucleotide polymorphisms used to build the genetic risk score for atrial fibrillation
Supplementary Table 12. Codes used in the UK Biobank study to identify atrial fibrillation cases
Supplementary Table 13. The numbers (percentages) of participants with missing covariates

Supplementary Figure 1. Flowchart for the selection of the analyzed study sample from the UK Biobank Study

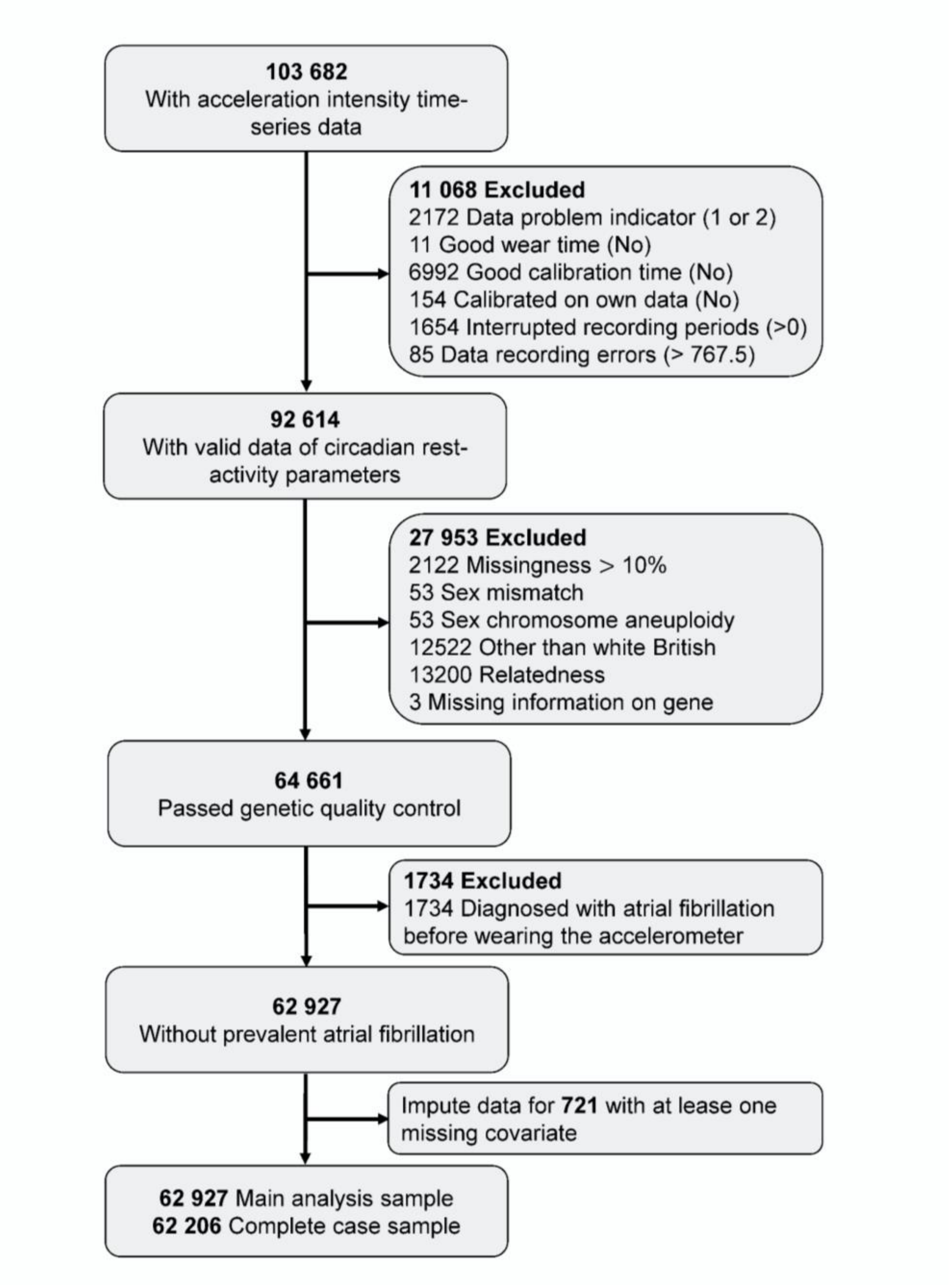

Supplementary Figure 2. Acceleration level during seven-day monitoring with fitted curve for the circadian rest-activity characteristics among participants without and with incident atrial fibrillation

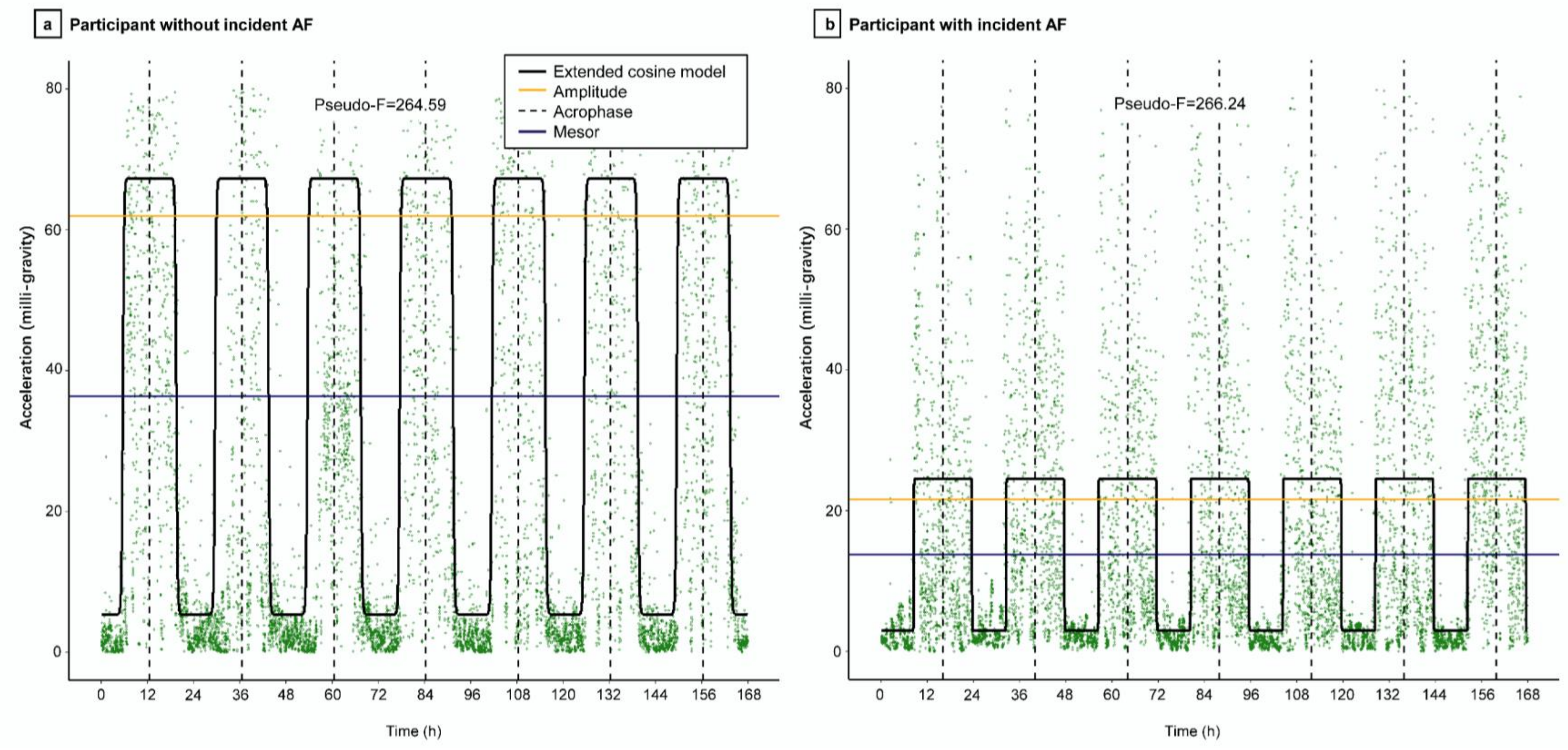

Abbreviation: AF, atrial fibrillation; Two participants, one without incident atrial fibrillation and the other one with incident atrial fibrillation, were selected from the study sample. a. The participant without incident atrial fibrillation had high amplitude and mesor as shown by the high overall level of activity and the high height of the fitted curve as well as advanced acrophase as shown by early peak activity of the fitted curve. b. The participant with incident atrial fibrillation had low amplitude and mesor as shown by the low overall level of activity and the dampened fitted curve as well as delayed acrophase as shown by late peak activity of the fitted curve.

Supplementary Figure 3. Standardized risk of incident atrial fibrillation according to genetic risk and amplitude

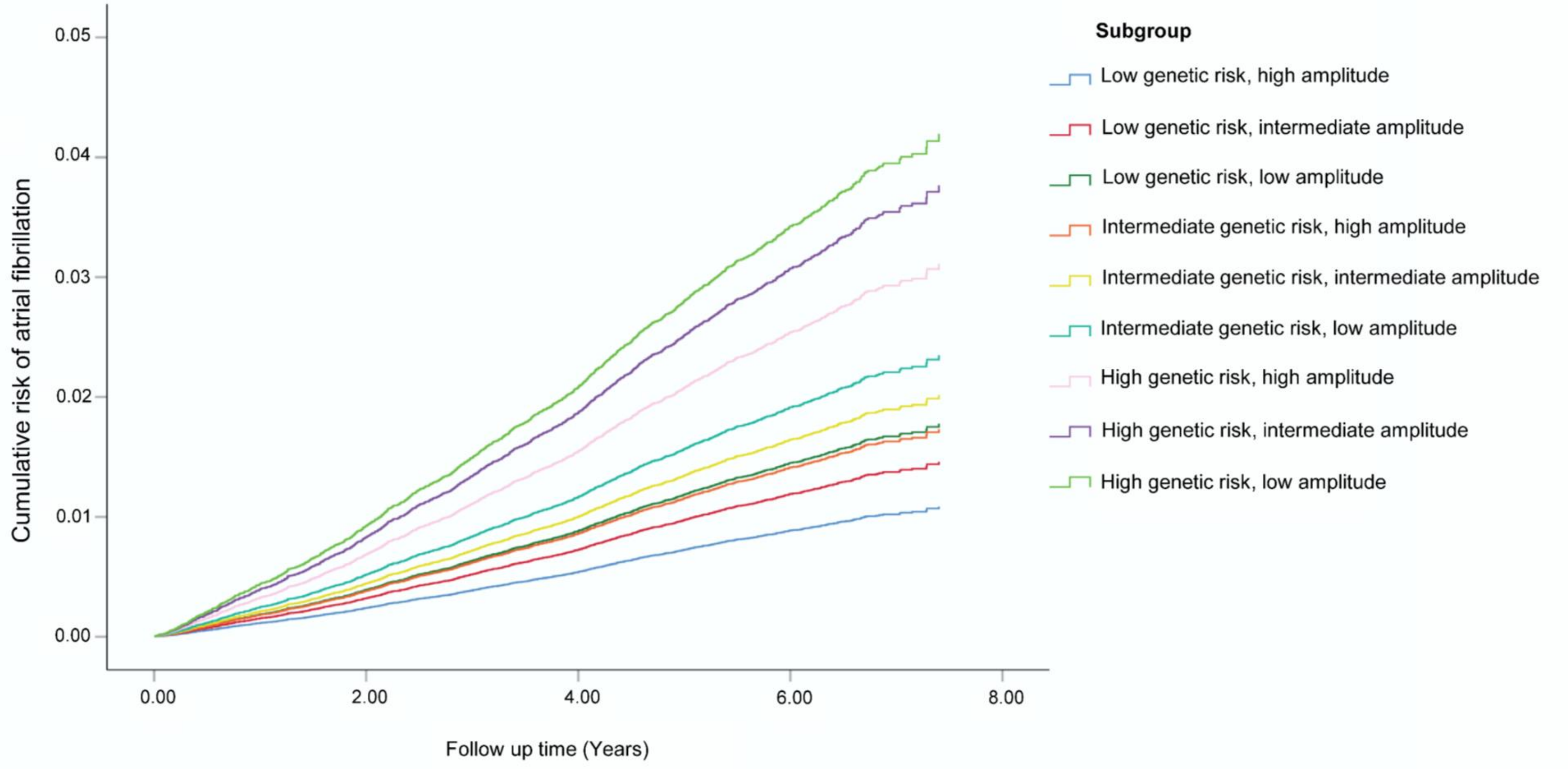

Abbreviation: AF, atrial fibrillation; BMI, body mass index. Cox proportional hazard models were adjusted for age, sex, Townsend deprivation index, recruitment centre, education level, season of accelerometer wear, BMI categories, healthy diet score, smoking status, alcohol intake, coffee consumption, tea consumption, hypertension, diabetes, dyslipidemia, sleep efficiency, sleep duration, genetic risk, and first 10 principal components of ancestry. Participants with prevalent AF were excluded.

Supplementary Figure 4. Standardized risk of incident atrial fibrillation according to genetic risk and acrophase

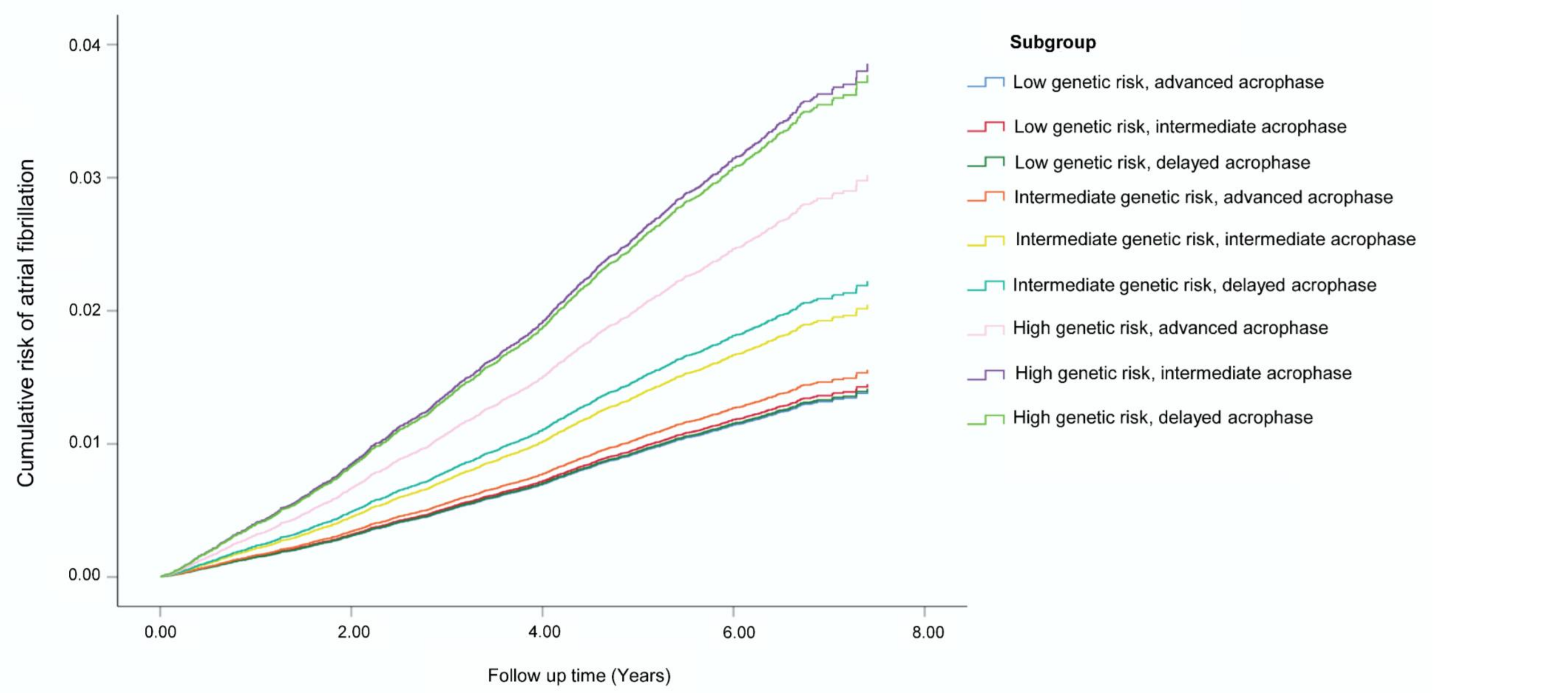

Abbreviation: AF, atrial fibrillation; BMI, body mass index. Cox proportional hazard models were adjusted for age, sex, Townsend deprivation index, recruitment centre, education level, season of accelerometer wear, BMI categories, healthy diet score, smoking status, alcohol intake, coffee consumption, tea consumption, hypertension, diabetes, dyslipidemia, sleep efficiency, sleep duration, genetic risk, and first 10 principal components of ancestry. Participants with prevalent AF were excluded.

Supplementary Figure 5. Standardized risk of incident atrial fibrillation according to genetic risk and pseudo-F

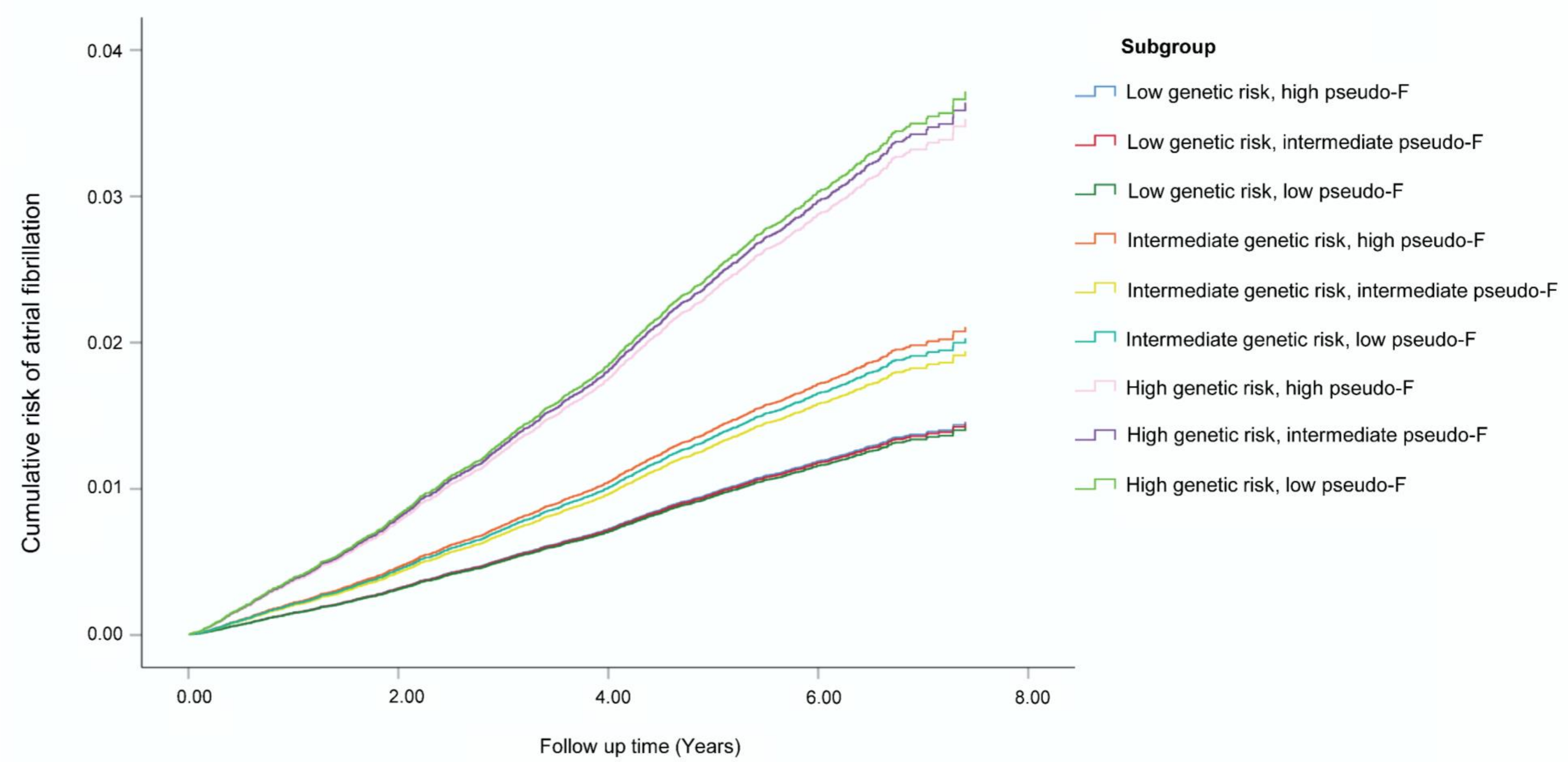

Abbreviation: AF, atrial fibrillation; BMI, body mass index. Cox proportional hazard models were adjusted for age, sex, Townsend deprivation index, recruitment centre, education level, season of accelerometer wear, BMI categories, healthy diet score, smoking status, alcohol intake, coffee consumption, tea consumption, hypertension, diabetes, dyslipidemia, sleep efficiency, sleep duration, genetic risk, and first 10 principal components of ancestry. Participants with prevalent AF were excluded.

Supplementary Figure 6. Standardized risk of incident atrial fibrillation according to genetic risk and mesor

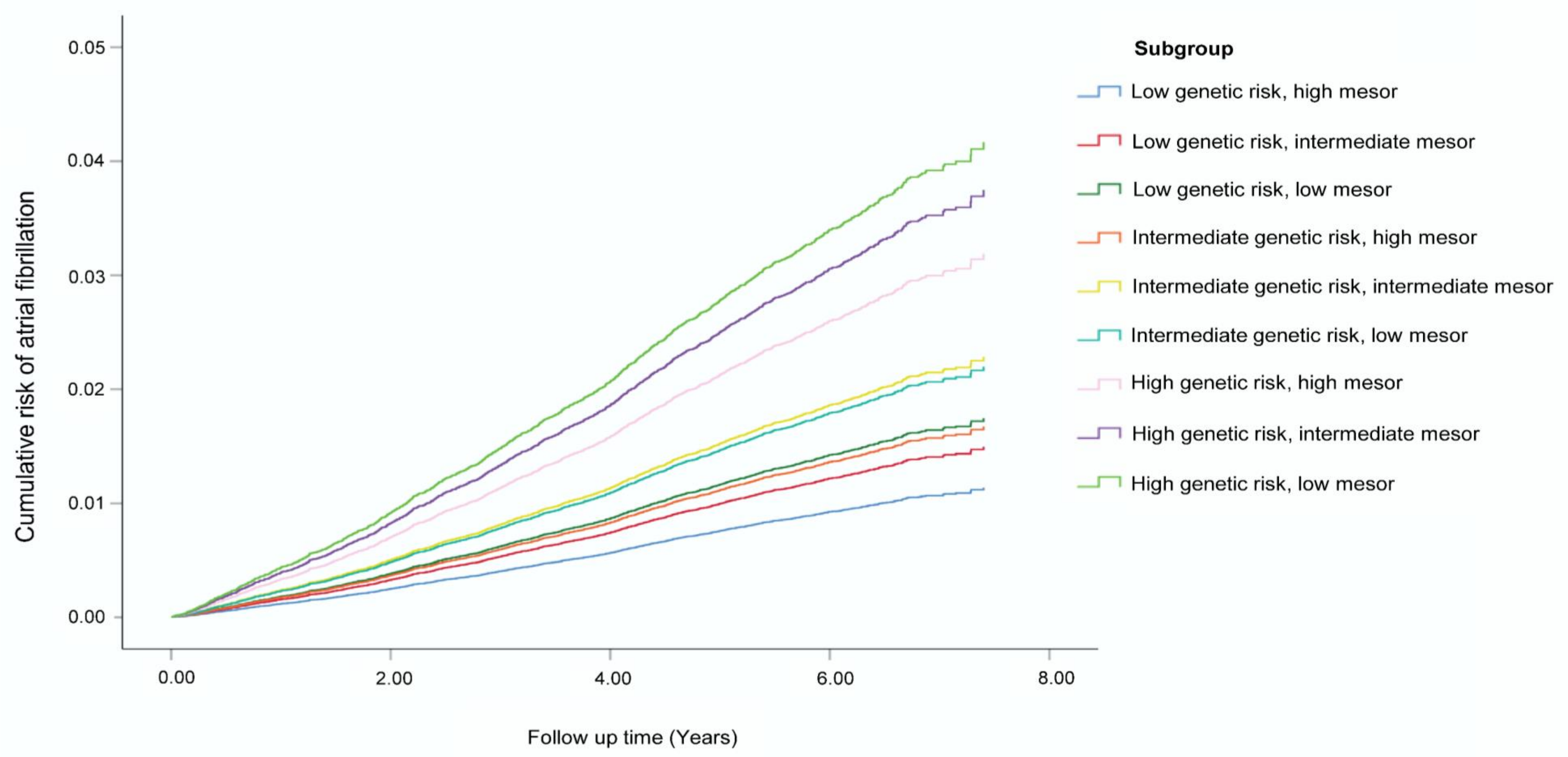

Abbreviation: AF, atrial fibrillation; BMI, body mass index. Cox proportional hazard models were adjusted for age, sex, Townsend deprivation index, recruitment centre, education level, season of accelerometer wear, BMI categories, healthy diet score, smoking status, alcohol intake, coffee consumption, tea consumption, hypertension, diabetes, dyslipidemia, sleep efficiency, sleep duration, genetic risk, and first 10 principal components of ancestry. Participants with prevalent AF were excluded.

Supplementary Figure 7. Timeline of covariates collection

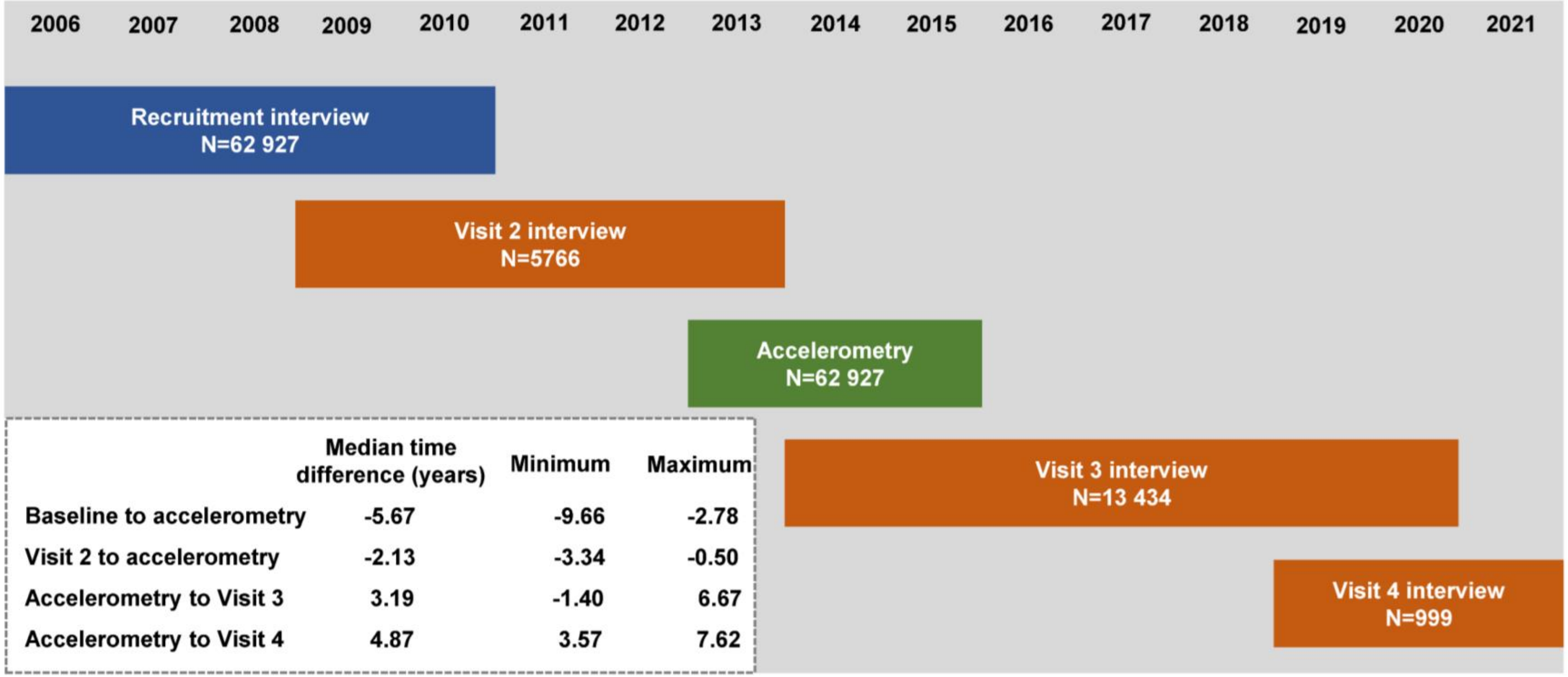

	Median time difference (years)	Minimum	Maximum
Baseline to accelerometry	-5.67	-9.66	-2.78
Visit 2 to accelerometry	-2.13	-3.34	-0.50
Accelerometry to Visit 3	3.19	-1.40	6.67
Accelerometry to Visit 4	4.87	3.57	7.62

Supplementary Figure 8. Associations between circadian rest-activity characteristics and incident atrial fibrillation

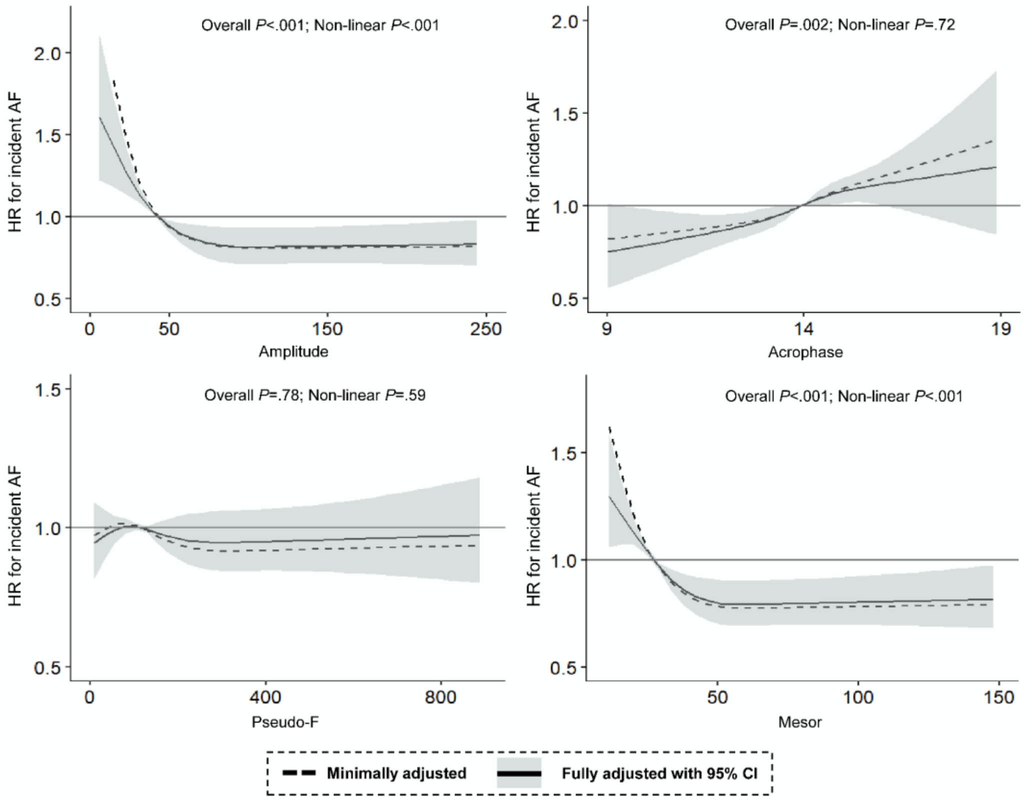

Abbreviation: AF, atrial fibrillation; BMI, body mass index; CI, confidence interval; HR, hazard ratio. In minimally adjusted models (Model 1, dash line), overall and non-linear testing analyses were adjusted for age and sex. In fully adjusted model (Model 4, solid line), overall and non-linear testing analyses were adjusted for age, sex, Townsend deprivation index, recruitment center, education level, season of accelerometer wear, BMI categories, healthy diet score, smoking status, alcohol intake, coffee consumption, tea consumption, hypertension, diabetes, dyslipidemia, sleep efficiency, and sleep duration, genetic risk, and first 10 principal components of ancestry. Shaded areas represent 95% confidence intervals (CIs). Participants with prevalent AF were excluded.

Supplementary Table 1. Risk of Incident atrial fibrillation according to circadian rest-activity characteristics

Circadian rest-activity characteristics	No. of AF cases/ person-years	Model 1<br>HR (95% CI); *P*	Model 2<br>HR (95% CI); *P*	Model 3<br>HR (95% CI); *P*	Model 4<br>HR (95% CI); *P*
Amplitude					
High (N=21137)	466/ 128 994	1[Reference]	1[Reference]	1[Reference]	1[Reference]
Intermediate (N=22656)	630/ 137 630	1.24 (1.10, 1.40); <0.001	1.24 (1.10, 1.40); <0.001	1.21 (1.07, 1.36); 0.002	1.22 (1.08, 1.38); 0.001[a]
Low (N=19134)	824/ 114 387	1.58 (1.41, 1.78); <0.001	1.58 (1.41, 1.77); <0.001	1.40 (1.25, 1.58); <0.001	1.41 (1.25, 1.58); <0.001[a]
P value for trend		*P<0.001*	*P<0.001*	*P<0.001*	*P<0.001*
Acrophase					
Advanced (N=12743)	421/ 76 728	1[Reference]	1[Reference]	1[Reference]	1[Reference]
Intermediate (N=20026)	635/ 121 026	1.20 (1.06, 1.35); 0.005	1.21 (1.07, 1.37); 0.003	1.23 (1.08, 1.39); 0.001	1.22 (1.08, 1.39); 0.001[a]
Delayed (N=30158)	864/ 183 256	1.23 (1.10, 1.39); <0.001	1.25 (1.11, 1.41); <0.001	1.24 (1.11, 1.40); <0.001	1.24 (1.10, 1.39); <0.001[a]
P value for trend		*P<0.001*	*P<0.001*	*P<0.001*	*P=0.001*
Pseudo-F					
High (N=15007)	439/ 91 306	1[Reference]	1[Reference]	1[Reference]	1[Reference]
Intermediate (N=20884)	645/ 126 353	1.01 (0.89, 1.14); 0.89	1.01 (0.90, 1.14); 0.87	1.00 (0.88, 1.12); 0.94	0.99 (0.88, 1.12); 0.85
Low (N=27036)	836/ 163 351	1.06 (0.94, 1.18); 0.37	1.05 (0.94, 1.18); 0.38	1.02 (0.91, 1.15); 0.70	1.01 (0.90, 1.13); 0.89
P value for trend		*P=0.32*	*P=0.35*	*P=0.66*	*P=0.84*
Mesor					
High (N=23755)	558/ 144 836	1[Reference]	1[Reference]	1[Reference]	1[Reference]
Intermediate (N=19756)	601/ 119 818	1.30 (1.16, 1.46); <0.001	1.30 (1.16, 1.46); <0.001	1.26 (1.12, 1.41); <0.001	1.26 (1.12, 1.41); <0.001[a]
Low (N=19416)	761/116 355	1.52 (1.37, 1.70); <0.001	1.52 (1.36, 1.69); <0.001	1.35 (1.21, 1.51); <0.001	1.36 (1.21, 1.52); <0.001[a]

P value for trend	*P<0.001*	*P<0.001*	*P<0.001*	*P<0.001*

Abbreviation: AF, atrial fibrillation; BMI, body mass index; CI, confidence interval; FDR, false-discovery rate; HR, hazard ratio.

Analyses were conducted using Cox proportional hazard models. **Model 1** was adjusted for age, sex, and first 10 principal components of ancestry. **Model 2** was adjusted as in model 1 and for Townsend deprivation index, recruitment centre, education level, and season of accelerometer wear. **Model 3** was adjusted as in model 2 and for BMI categories, healthy diet score, smoking status, alcohol intake, coffee consumption, tea consumption, hypertension, diabetes, and dyslipidemia. **Model 4** was adjusted as in model 3 and for sleep efficiency, sleep duration, and genetic risk. Participants with prevalent AF were excluded. [a] P values remained significant after multiple testing with FDR method.

Supplementary Table 2. Risk of Incident atrial fibrillation according to circadian rest-activity characteristics stratified by age categories

Circadian rest-activity characteristics	No. of AF cases/ person-years	Model 1<br>HR (95% CI); *P*	Model 2<br>HR (95% CI); *P*	Model 3<br>HR (95% CI); *P*	Model 4<br>HR (95% CI); *P*
**Age < 65 years**					
Amplitude					
High (N=12 982)	133/80 290	1[Reference]	1[Reference]	1[Reference]	1[Reference]
Intermediate (N=12 905)	179/79 628	1.43 (1.14, 1.79); 0.002	1.43 (1.14, 1.79); 0.002	1.38 (1.10, 1.73); 0.006	1.39 (1.11, 1.75); 0.004[a]
Low (N=9316)	161/57 238	1.61 (1.28, 2.03); <0.001	1.60 (1.27, 2.02); <0.001	1.40 (1.10, 1.77); 0.006	1.40 (1.10, 1.77); 0.006[a]
P value for trend		*P<0.001*	*P<0.001*	*P=0.005*	*P=0.006*
Acrophase					
Advanced (N=5763)	68/35 469	1[Reference]	1[Reference]	1[Reference]	1[Reference]
Intermediate (N=10 823)	162/66 660	1.50 (1.13, 1.99); 0.005	1.54 (1.16, 2.05); 0.003	1.56 (1.17, 2.07); 0.002	1.54 (1.16, 2.05); 0.003[a]
Delayed (N=18 617)	243/115 027	1.37 (1.04, 1.79); 0.02	1.40 (1.06, 1.83); 0.02	1.39 (1.06, 1.82); 0.02	1.36 (1.04, 1.79); 0.03[a]
P value for trend		*P=0.097*	*P=0.077*	*P=0.095*	*P=0.13*
Pseudo-F					
High (N=8349)	107/51 799	1[Reference]	1[Reference]	1[Reference]	1[Reference]
Intermediate (N=11 116)	140/68 586	0.93 (0.72, 1.19); 0.56	0.94 (0.73, 1.20); 0.61	0.92 (0.72, 1.19); 0.52	0.93 (0.72, 1.19); 0.55
Low (N=15 738)	226/96 772	1.00 (0.80, 1.27); 0.97	1.01 (0.80, 1.27); 0.96	0.97 (0.77, 1.22); 0.78	0.97 (0.77, 1.22); 0.79
P value for trend		*P=0.85*	*P=0.84*	*P=0.89*	*P=0.89*
Mesor					
High (N=13 826)	147/85 514	1[Reference]	1[Reference]	1[Reference]	1[Reference]
Intermediate (N=11 063)	155/68 283	1.38 (1.10, 1.73); 0.005	1.38 (1.10, 1.73); 0.005	1.31 (1.05, 1.65); 0.019	1.32 (1.05, 1.66); 0.017[a]

Low (N=10 314)	171/63 359	1.58 (1.27, 1.97); <0.001	1.57 (1.25, 1.95); <0.001	1.38 (1.10, 1.73); 0.006	1.39 (1.11, 1.75); 0.004[a]
P value for trend		*P<0.001*	*P<0.001*	*P=0.006*	*P=0.004*
**Age ≥ 65 years**					
Amplitude					
High (N=8155)	333/48704	1[Reference]	1[Reference]	1[Reference]	1[Reference]
Intermediate (N=9751)	451/58002	1.17 (1.02, 1.35); 0.027	1.17 (1.02, 1.35); 0.028	1.15 (0.99, 1.32); 0.06	1.16 (1.01, 1.34); 0.04
Low (N=9818)	663/57149	1.56 (1.37, 1.78); <0.001	1.56 (1.36, 1.78); <0.001	1.39 (1.22, 1.59); <0.001	1.40 (1.22, 1.60); <0.001[a]
P value for trend		*P<0.001*	*P<0.001*	*P<0.001*	*P<0.001*
Acrophase					
Advanced (N=6980)	353/41259	1[Reference]	1[Reference]	1[Reference]	1[Reference]
Intermediate (N=9203)	473/54366	1.13 (0.98, 1.29); 0.097	1.13 (0.99, 1.30); 0.077	1.15 (1.00, 1.32); 0.051	1.15 (1.00, 1.32); 0.051
Delayed (N=11541)	621/68229	1.21 (1.06, 1.38); 0.005	1.22 (1.07, 1.40); 0.003	1.22 (1.07, 1.39); 0.004	1.22 (1.06, 1.39); 0.004[a]
P value for trend		*P=0.005*	*P=0.003*	*P=0.004*	*P=0.005*
Pseudo-F					
High (N=6658)	332/39507	1[Reference]	1[Reference]	1[Reference]	1[Reference]
Intermediate (N=9768)	505/57767	1.04 (0.90, 1.19); 0.61	1.04 (0.90, 1.19); 0.62	1.02 (0.89, 1.17); 0.76	1.01 (0.88, 1.16); 0.86
Low (N=11298)	610/66580	1.08 (0.94, 1.23); 0.29	1.07 (0.94, 1.23); 0.32	1.04 (0.91, 1.19); 0.53	1.02 (0.89, 1.17); 0.74
P value for trend		*P=0.29*	*P=0.31*	*P=0.53*	*P=0.74*
Mesor					
High (N=9929)	411/59322	1[Reference]	1[Reference]	1[Reference]	1[Reference]
Intermediate (N=8693)	446/51536	1.27 (1.11, 1.46); <0.001	1.28 (1.12, 1.46); <0.001	1.23 (1.08, 1.41); 0.002	1.24 (1.08, 1.42); 0.002[a]
Low (N=9102)	590/52996	1.50 (1.32, 1.71); <0.001	1.50 (1.32, 1.70); <0.001	1.34 (1.17, 1.52); <0.001	1.34 (1.18, 1.53); <0.001[a]
P value for trend		*P<0.001*	*P<0.001*	*P<0.001*	*P<0.001*

Abbreviation: AF, atrial fibrillation; BMI, body mass index; CI, confidence interval; FDR, false-discovery rate; HR, hazard ratio.

Analyses were conducted using Cox proportional hazard models. **Model 1** was adjusted for age, sex, and first 10 principal components of ancestry. **Model 2** was adjusted as in model 1 and for Townsend deprivation

index, recruitment centre, education level, and season of accelerometer wear. **Model 3** was adjusted as in model 2 and for BMI categories, healthy diet score, smoking status, alcohol intake, coffee consumption, tea consumption, hypertension, diabetes, and dyslipidemia. **Model 4** was adjusted as in model 3 and for sleep efficiency, sleep duration, and genetic risk. Participants with prevalent AF were excluded. [a] P values remained significant after multiple testing with FDR method.

Supplementary Table 3. Risk of Incident atrial fibrillation according to circadian rest-activity characteristics stratified by sex categories

**Circadian rest-activity characteristics**	**No. of AF cases/ person-years**	**Model 1** HR (95% CI); *P*	**Model 2** HR (95% CI); *P*	**Model 3** HR (95% CI); *P*	**Model 4** HR (95% CI); *P*
**Females**					
Amplitude					
High (N=11281)	138/69281	1[Reference]	1[Reference]	1[Reference]	1[Reference]
Intermediate (N=13982)	276/85482	1.41 (1.15, 1.73); 0.001	1.41 (1.15, 1.73); 0.001	1.38 (1.13, 1.70); 0.002	1.39 (1.13, 1.70); 0.002[a]
Low (N=10060)	315/60829	1.92 (1.57, 2.35); <0.001	1.91 (1.56, 2.34); <0.001	1.69 (1.37, 2.08); <0.001	1.70 (1.38, 2.09); <0.001[a]
P value for trend		*P<0.001*	*P<0.001*	*P<0.001*	*P<0.001*
Acrophase					
Advanced (N=5926)	121/36029	1[Reference]	1[Reference]	1[Reference]	1[Reference]
Intermediate (N=11721)	245/71476	1.14 (0.91, 1.41); 0.25	1.15 (0.92, 1.43); 0.21	1.18 (0.95, 1.47); 0.14	1.19 (0.95, 1.48); 0.13
Delayed (N=17676)	363/108088	1.28 (1.04, 1.58); 0.02	1.28 (1.04, 1.58); 0.02	1.29 (1.05, 1.59); 0.02	1.29 (1.04, 1.58); 0.02[a]
P value for trend		*P=0.013*	*P=0.015*	*P=0.017*	*P=0.021*
Pseudo-F					
High (N=9112)	184/55827	1[Reference]	1[Reference]	1[Reference]	1[Reference]
Intermediate (N=12187)	256/74271	1.02 (0.85, 1.23); 0.83	1.03 (0.85, 1.24); 0.80	1.01 (0.84, 1.22); 0.93	1.02 (0.84, 1.24); 0.83
Low (N=14024)	289/85494	1.10 (0.91, 1.32); 0.32	1.09 (0.91, 1.31); 0.35	1.05 (0.87, 1.26); 0.62	1.05 (0.87, 1.27); 0.59
P value for trend		*P=0.29*	*P=0.34*	*P=0.60*	*P=0.58*
Mesor					
High (N=12815)	181/78593	1[Reference]	1[Reference]	1[Reference]	1[Reference]
Intermediate (N=11972)	255/73133	1.39 (1.14, 1.68); <0.001	1.38 (1.14, 1.67); <0.001	1.34 (1.11, 1.63); 0.003	1.35 (1.12, 1.64); 0.002[a]

Low (N=10536)	293/63867	1.70 (1.41, 2.05); <0.001	1.68 (1.40, 2.03); <0.001	1.49 (1.23, 1.81); <0.001	1.51 (1.25, 1.83); <0.001[a]
P value for trend		*P<0.001*	*P<0.001*	*P<0.001*	*P<0.001*
**Males**					
Amplitude					
High (N=9856)	328/59713	1[Reference]	1[Reference]	1[Reference]	1[Reference]
Intermediate (N=8674)	354/52147	1.17 (1.01, 1.36); 0.041	1.17 (1.01, 1.36); 0.041	1.14 (0.98, 1.32); 0.093	1.16 (0.99, 1.34); 0.06
Low (N=9074)	509/53557	1.44 (1.25, 1.65); <0.001	1.43 (1.25, 1.65); <0.001	1.28 (1.11, 1.48); <0.001	1.29 (1.11, 1.48); <0.001[a]
P value for trend		*P<0.001*	*P<0.001*	*P<0.001*	*P<0.001*
Acrophase					
Advanced (N=6817)	300/40699	1[Reference]	1[Reference]	1[Reference]	1[Reference]
Intermediate (N=8305)	390/49550	1.23 (1.06, 1.43); 0.008	1.25 (1.07, 1.45); 0.004	1.26 (1.08, 1.47); 0.003	1.25 (1.08, 1.46); 0.003[a]
Delayed (N=12482)	501/75168	1.19 (1.03, 1.38); 0.018	1.22 (1.05, 1.41); 0.008	1.21 (1.05, 1.40); 0.010	1.20 (1.04, 1.39); 0.014[a]
P value for trend		*P=0.03*	*P=0.01*	*P=0.02*	*P=0.03*
Pseudo-F					
High (N=5895)	255/35478	1[Reference]	1[Reference]	1[Reference]	1[Reference]
Intermediate (N=8697)	389/52081	1.00 (0.86, 1.17); 0.98	1.00 (0.86, 1.18); 0.97	0.99 (0.84, 1.16); 0.87	0.97 (0.83, 1.14); 0.71
Low (N=13012)	547/77857	1.03 (0.88, 1.19); 0.74	1.03 (0.89, 1.19); 0.72	1.00 (0.86, 1.16); 0.98	0.98 (0.84, 1.14); 0.77
P value for trend		*P=0.71*	*P=0.69*	*P=0.94*	*P=0.81*
Mesor					
High (N=10940)	377/66243	1[Reference]	1[Reference]	1[Reference]	1[Reference]
Intermediate (N=7784)	346/46685	1.27 (1.09, 1.46); 0.002	1.27 (1.10, 1.47); 0.001	1.22 (1.05, 1.41); 0.009	1.21 (1.05, 1.41); 0.01[a]
Low (N=8880)	468/52488	1.43 (1.25, 1.64); <0.001	1.43 (1.25, 1.64); <0.001	1.28 (1.11, 1.47); <0.001	1.28 (1.11, 1.47); <0.001[a]
P value for trend		*P<0.001*	*P<0.001*	*P<0.001*	*P<0.001*

Abbreviation: AF, atrial fibrillation; BMI, body mass index; CI, confidence interval; FDR, false-discovery rate; HR, hazard ratio.

Analyses were conducted using Cox proportional hazard models. **Model 1** was adjusted for age, sex, and first 10 principal components of ancestry. **Model 2** was adjusted as in model 1 and for Townsend deprivation

index, recruitment centre, education level, and season of accelerometer wear. **Model 3** was adjusted as in model 2 and for BMI categories, healthy diet score, smoking status, alcohol intake, coffee consumption, tea consumption, hypertension, diabetes, and dyslipidemia. **Model 4** was adjusted as in model 3 and for sleep efficiency, sleep duration, and genetic risk. Participants with prevalent AF were excluded. [a] P values remained significant after multiple testing with FDR method.

Supplementary Table 4. Risk of Incident atrial fibrillation according to genetic and circadian rest-activity characteristics

Subgroup	No. of AF cases/ person-years	Model 1 HR (95% CI); *P*	Model 2 HR (95% CI); *P*	Model 3 HR (95% CI); *P*	Model 4 HR (95% CI); *P*
**Amplitude**					
**Low genetic risk**					
High (N=7121)	89/43 571	1[Reference]	1[Reference]	1[Reference]	1[Reference]
Intermediate (N=7673)	134/46 802	1.38 (1.06, 1.81); 0.02	1.38 (1.06, 1.81); 0.02	1.35 (1.03, 1.76); 0.03	1.34 (1.03, 1.76); 0.03[a]
Low (N=6480)	187/38 993	1.85 (1.43, 2.38); <0.001	1.84 (1.43, 2.37); <0.001	1.65 (1.28, 2.13); <0.001	1.64 (1.27, 2.11); <0.001[a]
**Intermediate genetic risk**					
High (N=6976)	134/42 643	1.58 (1.21, 2.06); <0.001	1.58 (1.21, 2.07); <0.001	1.60 (1.22, 2.09); <0.001	1.59 (1.22, 2.08); <0.001[a]
Intermediate (N=7667)	183/46 750	1.90 (1.47, 2.44); <0.001	1.90 (1.47, 2.45); <0.001	1.85 (1.44, 2.39); <0.001	1.86 (1.44, 2.39); <0.001[a]
Low (N=6448)	244/38 578	2.49 (1.95, 3.17); <0.001	2.47 (1.94, 3.15); <0.001	2.18 (1.71, 2.78); <0.001	2.16 (1.69, 2.76); <0.001[a]
**High genetic risk**					
High (N=7040)	243/42 780	2.85 (2.24, 3.64); <0.001	2.85 (2.24, 3.64); <0.001	2.87 (2.25, 3.65); <0.001	2.87 (2.25, 3.66); <0.001[a]
Intermediate (N=7316)	313/44 078	3.54 (2.80, 4.48); <0.001	3.54 (2.80, 4.48); <0.001	3.47 (2.74, 4.40); <0.001	3.47 (2.74, 4.39); <0.001[a]
Low (N=6206)	393/36 816	4.35 (3.46, 5.48); <0.001	4.35 (3.46, 5.48); <0.001	3.90 (3.09, 4.92); <0.001	3.87 (3.07, 4.87); <0.001[a]
P value for trend		*P <0.001*	*P <0.001*	*P <0.001*	*P <0.001*
**Acrophase**					
**Low genetic risk**					
Advanced (N=4386)	109/26 489	1[Reference]	1[Reference]	1[Reference]	1[Reference]
Intermediate (N=6830)	133/41 374	0.99 (0.77, 1.28); 0.96	1.01 (0.78, 1.30); 0.94	1.03 (0.80, 1.33); 0.83	1.03 (0.80, 1.33); 0.80
Delayed (N=10 058)	168/61 503	0.98 (0.77, 1.25); 0.86	1.00 (0.78, 1.27); 0.99	1.00 (0.79, 1.28); 0.98	1.01 (0.79, 1.29); 0.94
**Intermediate genetic risk**					

Advanced (N=4291)	113/25 949	1.10 (0.85, 1.44); 0.46	1.11 (0.85, 1.44); 0.44	1.11 (0.86, 1.45); 0.42	1.11 (0.85, 1.45); 0.43
Intermediate (N=6613)	180/40 071	1.40 (1.11, 1.78); 0.005	1.43 (1.13, 1.81); 0.003	1.45 (1.14, 1.84); 0.002	1.46 (1.15, 1.85); 0.002[a]
Delayed (N=10 187)	268/61 950	1.56 (1.24, 1.94); <0.001	1.58 (1.26, 1.97); <0.001	1.57 (1.26, 1.97); <0.001	1.59 (1.27, 1.99); <0.001[a]
**High genetic risk**					
Advanced (N=4066)	199/24 289	2.11 (1.67, 2.67); <0.001	2.13 (1.69, 2.69); <0.001	2.16 (1.71, 2.73); <0.001	2.16 (1.71, 2.73); <0.001[a]
Intermediate (N=6583)	322/39 582	2.63 (2.11, 3.27); <0.001	2.67 (2.15, 3.32); <0.001	2.74 (2.20, 3.40); <0.001	2.75 (2.21, 3.43); <0.001[a]
Delayed (N=9913)	428/59 802	2.62 (2.12, 3.24); <0.001	2.66 (2.15, 3.29); <0.001	2.68 (2.17, 3.31); <0.001	2.69 (2.18, 3.33); <0.001[a]
P value for trend		*P <0.001*	*P <0.001*	*P <0.001*	*P <0.001*
**Pseudo-F**					
**Low genetic risk**					
High (N=5026)	96/30 676	1[Reference]	1[Reference]	1[Reference]	1[Reference]
Intermediate (N=7092)	141/43 111	1.00 (0.77, 1.29); 0.97	0.99 (0.77, 1.29); 0.96	0.99 (0.76, 1.28); 0.94	0.99 (0.76, 1.28); 0.94
Low (N=9156)	173/55 579	1.00 (0.78, 1.28); 0.98	1.00 (0.78, 1.28); >0.99	0.98 (0.76, 1.26); 0.87	0.97 (0.76, 1.25); 0.84
**Intermediate genetic risk**					
High (N=5082)	136/30 905	1.43 (1.10, 1.85); 0.008	1.43 (1.10, 1.86); 0.007	1.44 (1.11, 1.87); 0.006	1.44 (1.11, 1.88); 0.006[a]
Intermediate (N=6959)	181/42 269	1.34 (1.05, 1.72); 0.02	1.34 (1.05, 1.72); 0.02	1.33 (1.04, 1.70); 0.02	1.33 (1.04, 1.70); 0.02[a]
Low (N=9050)	244/54 797	1.44 (1.14, 1.82); 0.003	1.43 (1.13, 1.82); 0.003	1.40 (1.10, 1.77); 0.006	1.39 (1.10, 1.76); 0.006[a]
**High genetic risk**					
High (N=4899)	207/29 725	2.37 (1.86, 3.02); <0.001	2.36 (1.85, 3.01); <0.001	2.42 (1.90, 3.08); <0.001	2.42 (1.90, 3.08); <0.001[a]
Intermediate (N=6833)	323/40 972	2.49 (1.98, 3.12); <0.001	2.51 (2.00, 3.15); <0.001	2.50 (1.99, 3.14); <0.001	2.49 (1.99, 3.13); <0.001[a]
Low (N=8830)	419/52 976	2.62 (2.10, 3.27); <0.001	2.61 (2.09, 3.26); <0.001	2.57 (2.05, 3.20); <0.001	2.55 (2.04, 3.18); <0.001[a]
P value for trend		*P <0.001*	*P <0.001*	*P <0.001*	*P <0.001*
**Mesor**					
**Low genetic risk**					

High (N=8028)	112/49 093	1[Reference]	1[Reference]	1[Reference]	1[Reference]
Intermediate (N=6664)	125/40 575	1.36 (1.05, 1.75); 0.02	1.36 (1.05, 1.75); 0.02	1.32 (1.02, 1.70); 0.04	1.32 (1.02, 1.70); 0.04[a]
Low (N=6582)	173/39 698	1.71 (1.35, 2.17); <0.001	1.71 (1.35, 2.17); <0.001	1.53 (1.21, 1.95); <0.001	1.54 (1.21, 1.95); <0.001[a]
**Intermediate genetic risk**					
High (N=7875)	156/48 094	1.45 (1.14, 1.85); 0.003	1.46 (1.14, 1.86); 0.002	1.47 (1.15, 1.88); 0.002	1.47 (1.16, 1.88); 0.002[a]
Intermediate (N=6707)	189/40 769	2.08 (1.65, 2.63); <0.001	2.09 (1.65, 2.64); <0.001	2.01 (1.59, 2.54); <0.001	2.01 (1.59, 2.54); <0.001[a]
Low (N=6509)	216/39 107	2.19 (1.75, 2.76); <0.001	2.18 (1.74, 2.74); <0.001	1.93 (1.54, 2.43); <0.001	1.94 (1.54, 2.44); <0.001[a]
**High genetic risk**					
High (N=7852)	290/47 649	2.77 (2.23, 3.45); <0.001	2.78 (2.24, 3.46); <0.001	2.80 (2.25, 3.49); <0.001	2.81 (2.26, 3.49); <0.001[a]
Intermediate (N=6385)	287/38 474	3.39 (2.72, 4.22); <0.001	3.38 (2.72, 4.22); <0.001	3.30 (2.65, 4.11); <0.001	3.30 (2.65, 4.11); <0.001[a]
Low (N=6325)	372/37 551	4.09 (3.31, 5.05); <0.001	4.09 (3.31, 5.05); <0.001	3.67 (2.96, 4.53); <0.001	3.67 (2.97, 4.54); <0.001[a]
P value for trend		*P <0.001*	*P <0.001*	*P <0.001*	*P <0.001*

Abbreviation: AF, atrial fibrillation; BMI, body mass index; CI, confidence interval; FDR, false-discovery rate; HR, hazard ratio.

Analyses were conducted using Cox proportional hazard models. **Model 1** was adjusted for age, sex, and first 10 principal components of ancestry. **Model 2** was adjusted as in model 1 and for Townsend deprivation index, recruitment centre, education level, and season of accelerometer wear. **Model 3** was adjusted as in model 2 and for BMI categories, healthy diet score, smoking status, alcohol intake, coffee consumption, tea consumption, hypertension, diabetes, and dyslipidemia. **Model 4** was adjusted as in model 3 and for sleep efficiency and sleep duration. Participants with prevalent AF were excluded. [a] P values remained significant after multiple testing with FDR method.

Supplementary Table 5. Sensitivity analysis on the association of circadian rest-activity characteristics and genetic risk with Incident atrial fibrillation by using competing risk regression (Fine and Gray)

Subgroup	No. of AF cases/ person-years	**Model 1** HR (95% CI); *P*	**Model 2** HR (95% CI); *P*	**Model 3** HR (95% CI); *P*	**Model 4** HR (95% CI); *P*
**Amplitude**					
**Low genetic risk**					
High (N=7121)	89/43 571	1[Reference]	1[Reference]	1[Reference]	1[Reference]
Intermediate (N=7673)	134/46 802	1.38 (1.06, 1.80); 0.02	1.38 (1.06, 1.81); 0.02	1.35 (1.03, 1.76); 0.03	1.34 (1.03, 1.75); 0.03[a]
Low (N=6480)	187/38 993	1.83 (1.42, 2.35); <0.001	1.83 (1.42, 2.35); <0.001	1.64 (1.27, 2.11); <0.001	1.62 (1.26, 2.09); <0.001[a]
**Intermediate genetic risk**					
High (N=6976)	134/42 643	1.59 (1.21, 2.07); <0.001	1.59 (1.22, 2.08); <0.001	1.61 (1.23, 2.10); <0.001	1.61 (1.23, 2.10); <0.001[a]
Intermediate (N=7667)	183/46 750	1.90 (1.48, 2.45); <0.001	1.91 (1.48, 2.46); <0.001	1.86 (1.45, 2.40); <0.001	1.86 (1.45, 2.40); <0.001[a]
Low (N=6448)	244/38 578	2.47 (1.93, 3.14); <0.001	2.45 (1.92, 3.13); <0.001	2.17 (1.70, 2.78); <0.001	2.15 (1.69, 2.75); <0.001[a]
**High genetic risk**					
High (N=7040)	243/42 780	2.86 (2.24, 3.64); <0.001	2.86 (2.25, 3.65); <0.001	2.88 (2.26, 3.68); <0.001	2.88 (2.26, 3.67); <0.001[a]
Intermediate (N=7316)	313/44 078	3.54 (2.80, 4.48); <0.001	3.55 (2.80, 4.49); <0.001	3.48 (2.75, 4.40); <0.001	3.48 (2.75, 4.40); <0.001[a]
Low (N=6206)	393/36 816	4.31 (3.43, 5.43); <0.001	4.32 (3.43, 5.44); <0.001	3.89 (3.08, 4.90); <0.001	3.85 (3.05, 4.85); <0.001[a]
P value for trend		*P <0.001*	*P <0.001*	*P <0.001*	*P <0.001*
**Acrophase**					
**Low genetic risk**					
Advanced (N=4386)	109/26 489	1[Reference]	1[Reference]	1[Reference]	1[Reference]
Intermediate (N=6830)	133/41 374	1.00 (0.77, 1.29); 0.98	1.02 (0.79, 1.31); 0.91	1.04 (0.80, 1.33); 0.79	1.04 (0.81, 1.34); 0.76
Delayed (N=10 058)	168/61 503	0.98 (0.77, 1.25); 0.87	1.00 (0.79, 1.28); 0.99	1.01 (0.79, 1.29); 0.95	1.02 (0.80, 1.29); 0.90
**Intermediate genetic risk**					

Advanced (N=4291)	113/25 949	1.11 (0.85, 1.44); 0.45	1.12 (0.86, 1.45); 0.42	1.12 (0.86, 1.46); 0.40	1.12 (0.86, 1.46); 0.40
Intermediate (N=6613)	180/40 071	1.41 (1.11, 1.80); 0.004	1.44 (1.14, 1.83); 0.003	1.47 (1.16, 1.86); 0.002	1.48 (1.16, 1.88); 0.001[a]
Delayed (N=10 187)	268/61 950	1.56 (1.25, 1.95); <0.001	1.58 (1.27, 1.98); <0.001	1.59 (1.27, 1.98); <0.001	1.61 (1.28, 2.01); <0.001[a]
**High genetic risk**					
Advanced (N=4066)	199/24 289	2.12 (1.68, 2.68); <0.001	2.14 (1.69, 2.70); <0.001	2.18 (1.73, 2.75); <0.001	2.18 (1.72, 2.75); <0.001[a]
Intermediate (N=6583)	322/39 582	2.63 (2.12, 3.27); <0.001	2.68 (2.15, 3.33); <0.001	2.75 (2.21, 3.43); <0.001	2.77 (2.23, 3.45); <0.001[a]
Delayed (N=9913)	428/59 802	2.63 (2.13, 3.25); <0.001	2.67 (2.16, 3.30); <0.001	2.70 (2.18, 3.34); <0.001	2.72 (2.20, 3.37); <0.001[a]
P value for trend		*P <0.001*	*P <0.001*	*P <0.001*	*P <0.001*
**Pseudo-F**					
**Low genetic risk**					
High (N=5026)	96/30 676	1[Reference]	1[Reference]	1[Reference]	1[Reference]
Intermediate (N=7092)	141/43 111	1.00 (0.77, 1.30); >0.99	1.00 (0.77, 1.30); >0.99	1.00 (0.77, 1.29); 0.97	0.99 (0.77, 1.28); 0.95
Low (N=9156)	173/55 579	1.00 (0.78, 1.28); >0.99	1.00 (0.78, 1.29); 0.97	0.98 (0.77, 1.26); 0.89	0.97 (0.76, 1.25); 0.84
**Intermediate genetic risk**					
High (N=5082)	136/30 905	1.43 (1.10, 1.85); 0.008	1.44 (1.11, 1.87); 0.007	1.44 (1.11, 1.88); 0.006	1.45 (1.11, 1.88); 0.006[a]
Intermediate (N=6959)	181/42 269	1.36 (1.06, 1.74); 0.02	1.36 (1.06, 1.74); 0.02	1.34 (1.05, 1.72); 0.02	1.34 (1.05, 1.72); 0.02[a]
Low (N=9050)	244/54 797	1.45 (1.14, 1.83); 0.002	1.44 (1.14, 1.83); 0.002	1.41 (1.12, 1.79); 0.004	1.40 (1.11, 1.78); 0.005[a]
**High genetic risk**					
High (N=4899)	207/29 725	2.37 (1.86, 3.02); <0.001	2.37 (1.86, 3.02); <0.001	2.42 (1.90, 3.08); <0.001	2.42 (1.90, 3.08); <0.001[a]
Intermediate (N=6833)	323/40 972	2.50 (1.99, 3.14); <0.001	2.52 (2.01, 3.17); <0.001	2.52 (2.01, 3.16); <0.001	2.51 (2.00, 3.15); <0.001[a]
Low (N=8830)	419/52 976	2.63 (2.11, 3.28); <0.001	2.63 (2.10, 3.28); <0.001	2.59 (2.07, 3.23); <0.001	2.57 (2.06, 3.20); <0.001[a]
P value for trend		*P <0.001*	*P <0.001*	*P <0.001*	*P <0.001*
**Mesor**					
**Low genetic risk**					

High (N=8028)	112/49 093	1[Reference]	1[Reference]	1[Reference]	1[Reference]
Intermediate (N=6664)	125/40 575	1.35 (1.05, 1.74); 0.02	1.36 (1.05, 1.75); 0.02	1.31 (1.02, 1.69); 0.04	1.31 (1.01, 1.69); 0.04[a]
Low (N=6582)	173/39 698	1.69 (1.34, 2.15); <0.001	1.70 (1.34, 2.15); <0.001	1.52 (1.20, 1.93); <0.001	1.52 (1.20, 1.92); <0.001[a]
**Intermediate genetic risk**					
High (N=7875)	156/48 094	1.46 (1.14, 1.85); 0.002	1.46 (1.15, 1.86); 0.002	1.48 (1.16, 1.88); 0.002	1.48 (1.16, 1.88); 0.002[a]
Intermediate (N=6707)	189/40 769	2.08 (1.65, 2.63); <0.001	2.09 (1.66, 2.64); <0.001	2.01 (1.59, 2.54); <0.001	2.01 (1.59, 2.54); <0.001[a]
Low (N=6509)	216/39 107	2.17 (1.73, 2.73); <0.001	2.16 (1.72, 2.72); <0.001	1.93 (1.53, 2.42); <0.001	1.93 (1.53, 2.42); <0.001[a]
**High genetic risk**					
High (N=7852)	290/47 649	2.78 (2.23, 3.45); <0.001	2.79 (2.24, 3.47); <0.001	2.82 (2.27, 3.50); <0.001	2.82 (2.27, 3.50); <0.001[a]
Intermediate (N=6385)	287/38 474	3.39 (2.72, 4.21); <0.001	3.39 (2.72, 4.21); <0.001	3.30 (2.65, 4.10); <0.001	3.29 (2.65, 4.10); <0.001[a]
Low (N=6325)	372/37 551	4.04 (3.27, 5.00); <0.001	4.05 (3.28, 5.00); <0.001	3.64 (2.95, 4.50); <0.001	3.64 (2.95, 4.50); <0.001[a]
P value for trend		*P <0.001*	*P <0.001*	*P <0.001*	*P <0.001*

Abbreviation: AF, atrial fibrillation; BMI, body mass index; CI, confidence interval; FDR, false-discovery rate; HR, hazard ratio.

Analyses were conducted using Cox proportional hazard models. **Model 1** was adjusted for age, sex, and first 10 principal components of ancestry. **Model 2** was adjusted as in model 1 and for Townsend deprivation index, recruitment centre, education level, and season of accelerometer wear. **Model 3** was adjusted as in model 2 and for BMI categories, healthy diet score, smoking status, alcohol intake, coffee consumption, tea consumption, hypertension, diabetes, and dyslipidemia. **Model 4** was adjusted as in model 3 and for sleep efficiency and sleep duration. Participants with prevalent AF were excluded. [a] P values remained significant after multiple testing with FDR method.

Supplementary Table 6. Sensitivity analysis on the associations of circadian rest-activity characteristics and genetic risk with incident atrial fibrillation among participants without any missing covariate data

Subgroup	No. of AF cases/ person-years	Model 1<br>HR (95% CI); *P*	Model 2<br>HR (95% CI); *P*	Model 3<br>HR (95% CI); *P*	Model 4<br>HR (95% CI); *P*
**Amplitude**					
**Low genetic risk**					
High (N=7059)	89/43 195	1[Reference]	1[Reference]	1[Reference]	1[Reference]
Intermediate (N=7593)	133/46 312	1.37 (1.05, 1.79); 0.02	1.37 (1.05, 1.80); 0.02	1.34 (1.02, 1.75); 0.03	1.34 (1.02, 1.75); 0.04[a]
Low (N=6388)	183/38 428	1.82 (1.41, 2.34); <0.001	1.82 (1.41, 2.34); <0.001	1.63 (1.26, 2.10); <0.001	1.61 (1.25, 2.08); <0.001[a]
**Intermediate genetic risk**					
High (N=6909)	131/42 229	1.55 (1.18, 2.03); 0.001	1.55 (1.19, 2.03); 0.001	1.57 (1.20, 2.05); 0.001	1.56 (1.20, 2.05); 0.001[a]
Intermediate (N=7584)	182/46 238	1.89 (1.47, 2.44); <0.001	1.89 (1.47, 2.44); <0.001	1.85 (1.43, 2.38); <0.001	1.85 (1.44, 2.39); <0.001[a]
Low (N=6358)	240/38 011	2.47 (1.93, 3.15); <0.001	2.45 (1.92, 3.13); <0.001	2.16 (1.69, 2.76); <0.001	2.15 (1.68, 2.74); <0.001[a]
**High genetic risk**					
High (N=6975)	242/42 370	2.84 (2.22, 3.62); <0.001	2.84 (2.22, 3.62); <0.001	2.85 (2.24, 3.64); <0.001	2.86 (2.24, 3.64); <0.001[a]
Intermediate (N=7223)	305/43 519	3.47 (2.74, 4.39); <0.001	3.47 (2.74, 4.39); <0.001	3.41 (2.69, 4.32); <0.001	3.40 (2.69, 4.31); <0.001[a]
Low (N=6117)	389/36 283	4.34 (3.45, 5.47); <0.001	4.34 (3.45, 5.47); <0.001	3.89 (3.09, 4.91); <0.001	3.85 (3.06, 4.86); <0.001[a]
P value for trend		*P <0.001*	*P <0.001*	*P <0.001*	*P <0.001*
**Acrophase**					
**Low genetic risk**					
Advanced (N=4340)	107/26 214	1[Reference]	1[Reference]	1[Reference]	1[Reference]
Intermediate (N=6764)	132/40 967	1.01 (0.78, 1.30); 0.96	1.02 (0.79, 1.32); 0.86	1.04 (0.81, 1.34); 0.77	1.05 (0.81, 1.35); 0.74
Delayed (N=9936)	166/60 755	0.99 (0.77, 1.26); 0.92	1.01 (0.79, 1.29); 0.95	1.02 (0.80, 1.30); 0.91	1.02 (0.80, 1.30); 0.87
**Intermediate genetic risk**					

Advanced (N=4243)	111/25 656	1.11 (0.85, 1.45); 0.45	1.11 (0.85, 1.45); 0.43	1.12 (0.86, 1.46); 0.41	1.12 (0.86, 1.46); 0.42
Intermediate (N=6547)	179/39 658	1.43 (1.12, 1.81); 0.004	1.45 (1.14, 1.85); 0.002	1.48 (1.16, 1.88); 0.002	1.48 (1.17, 1.89); 0.001[a]
Delayed (N=10 061)	263/61 165	1.56 (1.25, 1.96); <0.001	1.58 (1.26, 1.99); <0.001	1.58 (1.26, 1.98); <0.001	1.59 (1.27, 2.00); <0.001[a]
**High genetic risk**					
Advanced (N=4014)	198/23 958	2.15 (1.70, 2.72); <0.001	2.17 (1.72, 2.75); <0.001	2.20 (1.74, 2.78); <0.001	2.20 (1.74, 2.78); <0.001[a]
Intermediate (N=6506)	317/39 127	2.64 (2.12, 3.29); <0.001	2.69 (2.16, 3.35); <0.001	2.75 (2.21, 3.43); <0.001	2.77 (2.22, 3.45); <0.001[a]
Delayed (N=9795)	421/59 087	2.63 (2.13, 3.26); <0.001	2.67 (2.16, 3.31); <0.001	2.69 (2.17, 3.33); <0.001	2.71 (2.19, 3.35); <0.001[a]
P value for trend		*P <0.001*	*P <0.001*	*P <0.001*	*P <0.001*
**F-Pseudo**					
**Low genetic risk**					
High (N=4970)	95/30 332	1[Reference]	1[Reference]	1[Reference]	1[Reference]
Intermediate (N=7015)	140/42 636	1.00 (0.77, 1.30); 0.99	1.00 (0.77, 1.29); 0.99	1.00 (0.77, 1.29); 0.97	1.00 (0.77, 1.29); 0.97
Low (N=9055)	170/54 967	0.99 (0.77, 1.27); 0.93	0.99 (0.77, 1.28); 0.96	0.97 (0.76, 1.25); 0.84	0.97 (0.75, 1.25); 0.80
**Intermediate genetic risk**					
High (N=5022)	134/30 533	1.42 (1.09, 1.85); 0.009	1.43 (1.10, 1.86); 0.008	1.44 (1.11, 1.87); 0.007	1.44 (1.11, 1.88); 0.006[a]
Intermediate (N=6875)	178/41 748	1.34 (1.04, 1.72); 0.02	1.34 (1.05, 1.72); 0.02	1.33 (1.04, 1.71); 0.03	1.33 (1.04, 1.71); 0.03[a]
Low (N=8954)	241/54 197	1.43 (1.13, 1.82); 0.003	1.43 (1.13, 1.81); 0.003	1.40 (1.10, 1.77); 0.006	1.39 (1.10, 1.76); 0.007[a]
**High genetic risk**					
High (N=4832)	204/29 332	2.37 (1.86, 3.02); <0.001	2.37 (1.85, 3.02); <0.001	2.42 (1.90, 3.09); <0.001	2.43 (1.90, 3.10); <0.001[a]
Intermediate (N=6747)	317/40 439	2.48 (1.97, 3.11); <0.001	2.50 (1.99, 3.15); <0.001	2.49 (1.98, 3.13); <0.001	2.49 (1.98, 3.13); <0.001[a]
Low (N=8736)	415/52 400	2.61 (2.09, 3.27); <0.001	2.60 (2.08, 3.26); <0.001	2.56 (2.05, 3.21); <0.001	2.55 (2.04, 3.19); <0.001[a]
P value for trend		*P <0.001*	*P <0.001*	*P <0.001*	*P <0.001*
**Mesor**					
**Low genetic risk**					

High (N=7956)	112/48 647	1[Reference]	1[Reference]	1[Reference]	1[Reference]
Intermediate (N=6597)	124/40 165	1.35 (1.04, 1.74); 0.02	1.35 (1.05, 1.75); 0.02	1.31 (1.01, 1.69); 0.04	1.31 (1.01, 1.69); 0.04[a]
Low (N=6487)	169/39 123	1.69 (1.33, 2.14); <0.001	1.69 (1.33, 2.14); <0.001	1.51 (1.19, 1.92); <0.001	1.52 (1.19, 1.93); <0.001[a]
**Intermediate genetic risk**					
High (N=7793)	153/47 584	1.43 (1.12, 1.83); 0.004	1.44 (1.13, 1.83); 0.004	1.45 (1.14, 1.85); 0.003	1.45 (1.14, 1.85); 0.003[a]
Intermediate (N=6629)	186/40 295	2.05 (1.63, 2.60); <0.001	2.06 (1.63, 2.61); <0.001	1.98 (1.57, 2.51); <0.001	1.99 (1.57, 2.51); <0.001[a]
Low (N=6429)	214/38 600	2.19 (1.74, 2.75); <0.001	2.17 (1.73, 2.73); <0.001	1.93 (1.53, 2.43); <0.001	1.93 (1.54, 2.43); <0.001[a]
**High genetic risk**					
High (N=7778)	288/47 184	2.75 (2.21, 3.42); <0.001	2.76 (2.22, 3.44); <0.001	2.79 (2.24, 3.47); <0.001	2.79 (2.25, 3.47); <0.001[a]
Intermediate (N=6309)	280/38 016	3.32 (2.67, 4.14); <0.001	3.32 (2.66, 4.13); <0.001	3.23 (2.59, 4.03); <0.001	3.23 (2.60, 4.03); <0.001[a]
Low (N=6228)	368/36 972	4.09 (3.31, 5.06); <0.001	4.09 (3.31, 5.06); <0.001	3.67 (2.97, 4.54); <0.001	3.67 (2.97, 4.54); <0.001[a]
P value for trend		*P <0.001*	*P <0.001*	*P <0.001*	*P <0.001*

Abbreviation: AF, atrial fibrillation; BMI, body mass index; CI, confidence interval; FDR, false-discovery rate; HR, hazard ratio.

Analyses were conducted using Cox proportional hazard models. **Model 1** was adjusted for age, sex, and first 10 principal components of ancestry. **Model 2** was adjusted as in model 1 and for Townsend deprivation index, recruitment centre, education level, and season of accelerometer wear. **Model 3** was adjusted as in model 2 and for BMI categories, healthy diet score, smoking status, alcohol intake, coffee consumption, tea consumption, hypertension, diabetes, and dyslipidemia. **Model 4** was adjusted as in model 3 and for sleep efficiency and sleep duration. Participants with prevalent AF were excluded. [a] P values remained significant after multiple testing with FDR method.

Supplementary Table 7. Sensitivity analysis on the associations of circadian rest-activity characteristics and genetic risk with incident atrial fibrillation by excluding events that occurred within the first year of follow-up

Subgroup	No. of AF cases/ person-years	Model 1 HR (95% CI); *P*	Model 2 HR (95% CI); *P*	Model 3 HR (95% CI); *P*	Model 4 HR (95% CI); *P*
**Amplitude**					
**Low genetic risk**					
High (N=7111)	79/43 564	1[Reference]	1[Reference]	1[Reference]	1[Reference]
Intermediate (N=7658)	119/46 795	1.38 (1.04, 1.83); 0.03	1.38 (1.04, 1.83); 0.03	1.35 (1.01, 1.79); 0.04	1.34 (1.01, 1.79); 0.04[a]
Low (N=6457)	164/38 981	1.83 (1.40, 2.39); <0.001	1.82 (1.39, 2.38); <0.001	1.64 (1.25, 2.14); <0.001	1.63 (1.24, 2.13); <0.001[a]
**Intermediate genetic risk**					
High (N=6962)	120/42 635	1.59 (1.20, 2.11); 0.001	1.60 (1.20, 2.12); 0.001	1.61 (1.21, 2.14); 0.001	1.61 (1.21, 2.14); 0.001[a]
Intermediate (N=7642)	158/46 738	1.84 (1.40, 2.41); <0.001	1.84 (1.41, 2.42); <0.001	1.80 (1.37, 2.36); <0.001	1.80 (1.38, 2.36); <0.001[a]
Low (N=6415)	211/38 561	2.43 (1.88, 3.15); <0.001	2.41 (1.86, 3.13); <0.001	2.13 (1.64, 2.77); <0.001	2.12 (1.63, 2.75); <0.001[a]
**High genetic risk**					
High (N=7001)	204/42 758	2.70 (2.08, 3.50); <0.001	2.70 (2.09, 3.51); <0.001	2.72 (2.10, 3.53); <0.001	2.72 (2.10, 3.53); <0.001[a]
Intermediate (N=7272)	269/44 053	3.43 (2.67, 4.41); <0.001	3.43 (2.66, 4.40); <0.001	3.36 (2.62, 4.32); <0.001	3.36 (2.61, 4.32); <0.001[a]
Low (N=6155)	342/36 793	4.29 (3.36, 5.48); <0.001	4.28 (3.35, 5.47); <0.001	3.86 (3.01, 4.93); <0.001	3.83 (2.99, 4.90); <0.001[a]
P value for trend		*P <0.001*	*P <0.001*	*P <0.001*	*P <0.001*
**Acrophase**					
**Low genetic risk**					
Advanced (N=4373)	96/26 482	1[Reference]	1[Reference]	1[Reference]	1[Reference]
Intermediate (N=6813)	116/41 365	0.98 (0.75, 1.29); 0.89	1.00 (0.76, 1.31); >0.99	1.02 (0.78, 1.33); 0.90	1.02 (0.78, 1.34); 0.87
Delayed (N=10 040)	150/61 493	0.99 (0.76, 1.28); 0.92	1.01 (0.78, 1.30); 0.95	1.02 (0.78, 1.31); 0.91	1.02 (0.79, 1.32); 0.87
**Intermediate genetic risk**					

Advanced (N=4277)	99/25 942	1.10 (0.83, 1.45); 0.51	1.10 (0.83, 1.46); 0.49	1.11 (0.84, 1.47); 0.48	1.10 (0.83, 1.46); 0.50
Intermediate (N=6590)	157/40 059	1.39 (1.08, 1.79); 0.01	1.42 (1.10, 1.83); 0.007	1.44 (1.11, 1.85); 0.005	1.44 (1.12, 1.86); 0.005[a]
Delayed (N=10 152)	233/61 933	1.53 (1.21, 1.94); <0.001	1.55 (1.22, 1.97); <0.001	1.55 (1.22, 1.97); <0.001	1.57 (1.23, 1.99); <0.001[a]
**High genetic risk**					
Advanced (N=4032)	165/24 270	1.99 (1.55, 2.56); <0.001	2.01 (1.56, 2.59); <0.001	2.04 (1.59, 2.63); <0.001	2.04 (1.59, 2.63); <0.001[a]
Intermediate (N=6540)	279/39 558	2.58 (2.05, 3.26); <0.001	2.63 (2.09, 3.32); <0.001	2.70 (2.14, 3.40); <0.001	2.71 (2.15, 3.43); <0.001[a]
Delayed (N=9856)	371/59 776	2.58 (2.06, 3.23); <0.001	2.62 (2.09, 3.28); <0.001	2.63 (2.10, 3.30); <0.001	2.65 (2.11, 3.32); <0.001[a]
P value for trend		*P <0.001*	*P <0.001*	*P <0.001*	*P <0.001*
**F-Pseudo**					
**Low genetic risk**					
High (N=5019)	89/30 672	1[Reference]	1[Reference]	1[Reference]	1[Reference]
Intermediate (N=7074)	123/43 103	0.94 (0.71, 1.23); 0.64	0.94 (0.71, 1.23); 0.63	0.93 (0.71, 1.23); 0.62	0.93 (0.71, 1.23); 0.63
Low (N=9133)	150/55 565	0.93 (0.72, 1.21); 0.61	0.94 (0.72, 1.22); 0.64	0.92 (0.71, 1.20); 0.54	0.92 (0.71, 1.19); 0.52
**Intermediate genetic risk**					
High (N=5060)	114/30 894	1.29 (0.98, 1.70); 0.07	1.30 (0.99, 1.72); 0.06	1.31 (0.99, 1.72); 0.06	1.31 (0.99, 1.73); 0.06
Intermediate (N=6933)	155/42 255	1.24 (0.96, 1.61); 0.11	1.24 (0.96, 1.61); 0.10	1.23 (0.95, 1.60); 0.12	1.23 (0.95, 1.60); 0.12
Low (N=9026)	220/54 784	1.40 (1.10, 1.79); 0.007	1.40 (1.09, 1.79); 0.008	1.37 (1.07, 1.75); 0.01	1.36 (1.06, 1.74); 0.02[a]
**High genetic risk**					
High (N=4871)	179/29 710	2.21 (1.72, 2.85); <0.001	2.21 (1.71, 2.85); <0.001	2.26 (1.75, 2.91); <0.001	2.26 (1.76, 2.92); <0.001[a]
Intermediate (N=6789)	279/40 951	2.32 (1.83, 2.95); <0.001	2.35 (1.85, 2.98); <0.001	2.34 (1.84, 2.97); <0.001	2.34 (1.84, 2.97); <0.001[a]
Low (N=8768)	357/52 944	2.42 (1.92, 3.05); <0.001	2.41 (1.91, 3.04); <0.001	2.37 (1.88, 3.00); <0.001	2.36 (1.87, 2.98); <0.001[a]
P value for trend		*P <0.001*	*P <0.001*	*P <0.001*	*P <0.001*
**Mesor**					
**Low genetic risk**					

High (N=8017)	101/49 085	1[Reference]	1[Reference]	1[Reference]	1[Reference]
Intermediate (N=6650)	111/40 570	1.33 (1.02, 1.74); 0.04	1.34 (1.02, 1.75); 0.04	1.30 (0.99, 1.70); 0.06	1.30 (0.99, 1.70); 0.06
Low (N=6559)	150/39 685	1.65 (1.28, 2.12); <0.001	1.65 (1.28, 2.12); <0.001	1.48 (1.15, 1.90); 0.003	1.48 (1.15, 1.91); 0.002[a]
**Intermediate genetic risk**					
High (N=7863)	144/48 087	1.49 (1.15, 1.92); 0.002	1.49 (1.16, 1.93); 0.002	1.51 (1.17, 1.94); 0.002	1.51 (1.17, 1.94); 0.002[a]
Intermediate (N=6677)	159/40 754	1.94 (1.51, 2.49); <0.001	1.94 (1.52, 2.50); <0.001	1.87 (1.46, 2.40); <0.001	1.87 (1.46, 2.41); <0.001[a]
Low (N=6479)	186/39 093	2.10 (1.65, 2.68); <0.001	2.08 (1.63, 2.66); <0.001	1.85 (1.45, 2.36); <0.001	1.85 (1.45, 2.37); <0.001[a]
**High genetic risk**					
High (N=7811)	249/47 625	2.64 (2.10, 3.33); <0.001	2.66 (2.11, 3.35); <0.001	2.68 (2.13, 3.38); <0.001	2.68 (2.13, 3.38); <0.001[a]
Intermediate (N=6346)	248/38 453	3.25 (2.58, 4.10); <0.001	3.25 (2.58, 4.09); <0.001	3.17 (2.51, 3.99); <0.001	3.17 (2.51, 3.99); <0.001[a]
Low (N=6271)	318/37 527	3.90 (3.12, 4.88); <0.001	3.89 (3.11, 4.87); <0.001	3.50 (2.79, 4.38); <0.001	3.50 (2.80, 4.39); <0.001[a]
P value for trend		*P <0.001*	*P <0.001*	*P <0.001*	*P <0.001*

Abbreviation: AF, atrial fibrillation; BMI, body mass index; CI, confidence interval; FDR, false-discovery rate; HR, hazard ratio.

Analyses were conducted using Cox proportional hazard models. **Model 1** was adjusted for age, sex, and first 10 principal components of ancestry. **Model 2** was adjusted as in model 1 and for Townsend deprivation index, recruitment centre, education level, and season of accelerometer wear. **Model 3** was adjusted as in model 2 and for BMI categories, healthy diet score, smoking status, alcohol intake, coffee consumption, tea consumption, hypertension, diabetes, and dyslipidemia. **Model 4** was adjusted as in model 3 and for sleep efficiency and sleep duration. Participants with prevalent AF were excluded. [a] P values remained significant after multiple testing with FDR method.

Supplementary Table 8. Sensitivity analysis on the associations of circadian rest-activity characteristics and genetic risk with atrial fibrillation by censoring up to Dec 31, 2019

Subgroup	No. of AF cases/ person-years	Model 1 HR (95% CI); *P*	Model 2 HR (95% CI); *P*	Model 3 HR (95% CI); *P*	Model 4 HR (95% CI); *P*
**Amplitude**					
**Low genetic risk**					
High (N=7111)	67/36 345	1[Reference]	1[Reference]	1[Reference]	1[Reference]
Intermediate (N=7658)	97/39 072	1.34 (1.00, 1.79); 0.047	1.35 (1.01, 1.80); 0.046	1.31 (0.98, 1.76); 0.07	1.31 (0.98, 1.75); 0.07
Low (N=6457)	127/32 628	1.71 (1.30, 2.25); <0.001	1.71 (1.30, 2.25); <0.001	1.54 (1.17, 2.03); 0.002	1.52 (1.15, 2.01); 0.003[a]
**Intermediate genetic risk**					
High (N=6962)	98/35 588	1.52 (1.14, 2.04); 0.004	1.53 (1.14, 2.04); 0.004	1.54 (1.15, 2.06); 0.004	1.54 (1.15, 2.05); 0.004[a]
Intermediate (N=7642)	124/39 034	1.79 (1.36, 2.36); <0.001	1.79 (1.36, 2.36); <0.001	1.76 (1.33, 2.31); <0.001	1.76 (1.33, 2.31); <0.001[a]
Low (N=6415)	164/32 271	2.32 (1.78, 3.02); <0.001	2.31 (1.77, 3.01); <0.001	2.05 (1.57, 2.67); <0.001	2.03 (1.55, 2.64); <0.001[a]
**High genetic risk**					
High (N=7001)	171/35 758	2.84 (2.19, 3.69); <0.001	2.84 (2.19, 3.69); <0.001	2.85 (2.20, 3.70); <0.001	2.85 (2.20, 3.70); <0.001[a]
Intermediate (N=7272)	232/36 863	3.62 (2.81, 4.66); <0.001	3.62 (2.81, 4.67); <0.001	3.56 (2.76, 4.58); <0.001	3.55 (2.76, 4.57); <0.001[a]
Low (N=6155)	279/30 904	4.20 (3.27, 5.38); <0.001	4.20 (3.27, 5.38); <0.001	3.79 (2.95, 4.87); <0.001	3.74 (2.92, 4.81); <0.001[a]
P value for trend		*P <0.001*	*P <0.001*	*P <0.001*	*P <0.001*
**Acrophase**					
**Low genetic risk**					
Advanced (N=4373)	81/22 142	1[Reference]	1[Reference]	1[Reference]	1[Reference]
Intermediate (N=6813)	90/34 521	0.94 (0.71, 1.24); 0.66	0.96 (0.72, 1.26); 0.75	0.97 (0.73, 1.28); 0.82	0.98 (0.74, 1.29); 0.86
Delayed (N=10 040)	120/51 382	0.94 (0.72, 1.22); 0.64	0.96 (0.74, 1.25); 0.77	0.97 (0.74, 1.26); 0.79	0.97 (0.75, 1.27); 0.84
**Intermediate genetic risk**					

Advanced (N=4277)	78/21 679	1.04 (0.78, 1.39); 0.77	1.05 (0.79, 1.40); 0.75	1.05 (0.79, 1.40); 0.73	1.05 (0.79, 1.40); 0.75
Intermediate (N=6590)	126/33 458	1.36 (1.05, 1.76); 0.02	1.39 (1.07, 1.80); 0.01	1.41 (1.09, 1.82); 0.01	1.42 (1.09, 1.84); 0.008[a]
Delayed (N=10 152)	182/51 756	1.47 (1.15, 1.87); 0.002	1.50 (1.17, 1.91); 0.001	1.49 (1.17, 1.90); 0.001	1.50 (1.18, 1.92); 0.001[a]
**High genetic risk**					
Advanced (N=4032)	139/20 334	2.13 (1.66, 2.74); <0.001	2.15 (1.67, 2.76); <0.001	2.17 (1.69, 2.80); <0.001	2.17 (1.69, 2.79); <0.001[a]
Intermediate (N=6540)	234/33 128	2.64 (2.08, 3.33); <0.001	2.68 (2.12, 3.39); <0.001	2.74 (2.16, 3.46); <0.001	2.76 (2.18, 3.49); <0.001[a]
Delayed (N=9856)	309/50 063	2.61 (2.08, 3.28); <0.001	2.66 (2.12, 3.34); <0.001	2.67 (2.12, 3.35); <0.001	2.69 (2.14, 3.38); <0.001[a]
P value for trend		*P <0.001*	*P <0.001*	*P <0.001*	*P <0.001*
**F-Pseudo**					
**Low genetic risk**					
High (N=5019)	68/25 650	1[Reference]	1[Reference]	1[Reference]	1[Reference]
Intermediate (N=7074)	96/35 997	1.03 (0.77, 1.38); 0.83	1.03 (0.77, 1.38); 0.85	1.03 (0.77, 1.38); 0.86	1.02 (0.77, 1.37); 0.87
Low (N=9133)	127/46 398	1.10 (0.84, 1.46); 0.49	1.11 (0.84, 1.46); 0.47	1.09 (0.82, 1.43); 0.56	1.08 (0.82, 1.42); 0.60
**Intermediate genetic risk**					
High (N=5060)	90/25 836	1.50 (1.12, 2.01); 0.006	1.51 (1.13, 2.02); 0.006	1.52 (1.13, 2.04); 0.005	1.52 (1.14, 2.04); 0.005[a]
Intermediate (N=6933)	120/35 291	1.39 (1.05, 1.83); 0.02	1.39 (1.05, 1.83); 0.02	1.37 (1.04, 1.81); 0.03	1.37 (1.04, 1.81); 0.03[a]
Low (N=9026)	176/45 766	1.50 (1.15, 1.96); 0.003	1.50 (1.15, 1.95); 0.003	1.46 (1.12, 1.91); 0.005	1.45 (1.11, 1.90); 0.006[a]
**High genetic risk**					
High (N=4871)	153/24 913	2.64 (2.01, 3.45); <0.001	2.62 (2.00, 3.43); <0.001	2.67 (2.04, 3.50); <0.001	2.68 (2.05, 3.51); <0.001[a]
Intermediate (N=6789)	232/34 275	2.72 (2.11, 3.51); <0.001	2.74 (2.13, 3.54); <0.001	2.73 (2.11, 3.52); <0.001	2.72 (2.11, 3.51); <0.001[a]
Low (N=8768)	297/44 338	2.86 (2.23, 3.66); <0.001	2.84 (2.22, 3.65); <0.001	2.80 (2.18, 3.59); <0.001	2.78 (2.16, 3.56); <0.001[a]
P value for trend		*P <0.001*	*P <0.001*	*P <0.001*	*P <0.001*
**Mesor**					
**Low genetic risk**					

High (N=8017)	82/40 959	1[Reference]	1[Reference]	1[Reference]	1[Reference]
Intermediate (N=6650)	96/33 887	1.44 (1.09, 1.90); 0.009	1.45 (1.10, 1.91); 0.009	1.41 (1.07, 1.85); 0.02	1.40 (1.07, 1.85); 0.02[a]
Low (N=6559)	113/33 198	1.62 (1.24, 2.11); <0.001	1.62 (1.25, 2.11); <0.001	1.46 (1.12, 1.91); 0.005	1.46 (1.12, 1.91); 0.005[a]
**Intermediate genetic risk**					
High (N=7863)	115/40 141	1.42 (1.09, 1.86); 0.01	1.42 (1.09, 1.86); 0.01	1.44 (1.10, 1.88); 0.008	1.44 (1.10, 1.88); 0.008[a]
Intermediate (N=6677)	127/34 057	2.09 (1.61, 2.70); <0.001	2.09 (1.62, 2.71); <0.001	2.02 (1.56, 2.61); <0.001	2.02 (1.56, 2.61); <0.001[a]
Low (N=6479)	144/32 695	2.13 (1.65, 2.74); <0.001	2.12 (1.65, 2.72); <0.001	1.89 (1.47, 2.44); <0.001	1.89 (1.47, 2.44); <0.001[a]
**High genetic risk**					
High (N=7811)	208/39 834	2.86 (2.25, 3.63); <0.001	2.87 (2.26, 3.64); <0.001	2.89 (2.27, 3.66); <0.001	2.89 (2.28, 3.67); <0.001[a]
Intermediate (N=6346)	210/32 206	3.55 (2.79, 4.50); <0.001	3.54 (2.79, 4.50); <0.001	3.46 (2.72, 4.39); <0.001	3.45 (2.72, 4.39); <0.001[a]
Low (N=6271)	264/31 485	4.19 (3.33, 5.28); <0.001	4.19 (3.33, 5.29); <0.001	3.79 (3.00, 4.78); <0.001	3.79 (3.00, 4.78); <0.001[a]
P value for trend		*P <0.001*	*P <0.001*	*P <0.001*	*P <0.001*

Abbreviation: AF, atrial fibrillation; BMI, body mass index; CI, confidence interval; FDR, false-discovery rate; HR, hazard ratio.

Analyses were conducted using Cox proportional hazard models. **Model 1** was adjusted for age, sex, and first 10 principal components of ancestry. **Model 2** was adjusted as in model 1 and for Townsend deprivation index, recruitment centre, education level, and season of accelerometer wear. **Model 3** was adjusted as in model 2 and for BMI categories, healthy diet score, smoking status, alcohol intake, coffee consumption, tea consumption, hypertension, diabetes, and dyslipidemia. **Model 4** was adjusted as in model 3 and for sleep efficiency and sleep duration. Participants with prevalent AF were excluded. [a] P values remained significant after multiple testing with FDR method.

Supplementary Table 9. Sensitivity analysis on the associations of circadian rest-activity characteristics and genetic risk with atrial fibrillation by excluding those reporting a history of shift work

**Subgroup**	**No. of AF cases/ person-years**	**Model 1** HR (95% CI); *P*	**Model 2** HR (95% CI); *P*	**Model 3** HR (95% CI); *P*	**Model 4** HR (95% CI); *P*
**Amplitude**					
**Low genetic risk**					
High (N=5471)	69/33 474	1[Reference]	1[Reference]	1[Reference]	1[Reference]
Intermediate (N=6021)	108/36 729	1.40 (1.03, 1.89); 0.03	1.40 (1.04, 1.90); 0.03	1.36 (1.01, 1.84); 0.046	1.36 (1.00, 1.84); 0.048[a]
Low (N=5150)	150/30 943	1.86 (1.40, 2.48); <0.001	1.85 (1.39, 2.47); <0.001	1.66 (1.25, 2.22); <0.001	1.65 (1.24, 2.19); <0.001[a]
**Intermediate genetic risk**					
High (N=5333)	109/32 661	1.64 (1.22, 2.22); 0.001	1.65 (1.22, 2.23); 0.001	1.65 (1.22, 2.23); 0.001	1.65 (1.22, 2.23); 0.001[a]
Intermediate (N=6034)	155/36 798	2.01 (1.51, 2.66); <0.001	2.00 (1.51, 2.66); <0.001	1.96 (1.47, 2.60); <0.001	1.96 (1.48, 2.61); <0.001[a]
Low (N=5089)	200/30 430	2.58 (1.96, 3.40); <0.001	2.56 (1.94, 3.37); <0.001	2.26 (1.71, 2.97); <0.001	2.24 (1.70, 2.95); <0.001[a]
**High genetic risk**					
High (N=5392)	181/32 811	2.72 (2.06, 3.59); <0.001	2.71 (2.05, 3.58); <0.001	2.71 (2.06, 3.58); <0.001	2.71 (2.06, 3.58); <0.001[a]
Intermediate (N=5840)	242/35 241	3.37 (2.58, 4.40); <0.001	3.37 (2.58, 4.40); <0.001	3.31 (2.53, 4.33); <0.001	3.30 (2.53, 4.32); <0.001[a]
Low (N=4881)	316/28 887	4.44 (3.42, 5.76); <0.001	4.42 (3.40, 5.73); <0.001	3.97 (3.05, 5.16); <0.001	3.93 (3.02, 5.11); <0.001[a]
P value for trend		*P <0.001*	*P <0.001*	*P <0.001*	*P <0.001*
**Acrophase**					
**Low genetic risk**					
Advanced (N=3369)	88/20 331	1[Reference]	1[Reference]	1[Reference]	1[Reference]
Intermediate (N=5379)	109/32 553	0.97 (0.73, 1.28); 0.81	0.98 (0.74, 1.30); 0.88	1.00 (0.76, 1.33); >0.99	1.01 (0.76, 1.34); 0.96
Delayed (N=7894)	130/48 282	0.89 (0.68, 1.17); 0.42	0.91 (0.69, 1.19); 0.48	0.92 (0.70, 1.21); 0.54	0.93 (0.70, 1.21); 0.57
**Intermediate genetic risk**					

Advanced (N=3300)	91/19 994	1.07 (0.80, 1.43); 0.65	1.07 (0.80, 1.43); 0.66	1.07 (0.80, 1.43); 0.65	1.07 (0.80, 1.43); 0.65
Intermediate (N=5176)	150/31 357	1.40 (1.07, 1.82); 0.01	1.42 (1.09, 1.84); 0.01	1.45 (1.11, 1.89); 0.006	1.46 (1.12, 1.90); 0.005[a]
Delayed (N=7980)	223/48 539	1.54 (1.20, 1.97); <0.001	1.55 (1.21, 1.99); <0.001	1.56 (1.22, 2.00); <0.001	1.58 (1.23, 2.02); <0.001[a]
**High genetic risk**					
Advanced (N=3103)	152/18 549	1.96 (1.51, 2.55); <0.001	1.97 (1.51, 2.56); <0.001	2.00 (1.54, 2.60); <0.001	1.99 (1.53, 2.59); <0.001[a]
Intermediate (N=5265)	256/31 664	2.46 (1.93, 3.14); <0.001	2.50 (1.96, 3.18); <0.001	2.58 (2.02, 3.29); <0.001	2.60 (2.04, 3.32); <0.001[a]
Delayed (N=7745)	331/46 756	2.40 (1.89, 3.04); <0.001	2.42 (1.91, 3.07); <0.001	2.45 (1.93, 3.10); <0.001	2.47 (1.95, 3.12); <0.001[a]
P value for trend		*P <0.001*	*P <0.001*	*P <0.001*	*P <0.001*
**F-Pseudo**					
**Low genetic risk**					
High (N=4036)	72/24 626	1[Reference]	1[Reference]	1[Reference]	1[Reference]
Intermediate (N=5615)	108/34 119	1.02 (0.76, 1.38); 0.88	1.02 (0.76, 1.37); 0.90	1.02 (0.76, 1.37); 0.91	1.02 (0.76, 1.37); 0.91
Low (N=6991)	147/42 402	1.17 (0.88, 1.55); 0.27	1.18 (0.89, 1.56); 0.26	1.15 (0.87, 1.53); 0.32	1.15 (0.86, 1.52); 0.34
**Intermediate genetic risk**					
High (N=4031)	111/24 594	1.55 (1.15, 2.08); 0.004	1.55 (1.16, 2.09); 0.004	1.56 (1.16, 2.10); 0.003	1.56 (1.16, 2.10); 0.003[a]
Intermediate (N=5559)	153/33 753	1.50 (1.14, 1.99); 0.004	1.50 (1.13, 1.99); 0.005	1.49 (1.13, 1.97); 0.005	1.49 (1.13, 1.98); 0.005[a]
Low (N=6866)	200/41 542	1.64 (1.25, 2.15); <0.001	1.63 (1.24, 2.13); <0.001	1.60 (1.22, 2.09); 0.001	1.59 (1.21, 2.08); 0.001[a]
**High genetic risk**					
High (N=3925)	175/23 808	2.66 (2.02, 3.50); <0.001	2.64 (2.01, 3.48); <0.001	2.70 (2.05, 3.55); <0.001	2.70 (2.05, 3.55); <0.001[a]
Intermediate (N=5464)	248/32 762	2.55 (1.96, 3.31); <0.001	2.56 (1.97, 3.32); <0.001	2.55 (1.96, 3.31); <0.001	2.54 (1.96, 3.31); <0.001[a]
Low (N=6724)	316/40 369	2.71 (2.10, 3.50); <0.001	2.70 (2.09, 3.49); <0.001	2.67 (2.07, 3.45); <0.001	2.66 (2.06, 3.44); <0.001[a]
P value for trend		*P <0.001*	*P <0.001*	*P <0.001*	*P <0.001*
**Mesor**					
**Low genetic risk**					

High (N=6199)	90/37 871	1[Reference]	1[Reference]	1[Reference]	1[Reference]
Intermediate (N=5222)	102/31 796	1.34 (1.01, 1.78); 0.04	1.34 (1.01, 1.78); 0.04	1.29 (.97, 1.72); 0.08	1.29 (.97, 1.71); 0.08
Low (N=5221)	135/31 480	1.63 (1.25, 2.12); <0.001	1.62 (1.24, 2.12); <0.001	1.47 (1.12, 1.92); 0.005	1.47(1.12, 1.92); 0.005[a]
**Intermediate genetic risk**					
High (N=6068)	129/37 104	1.47 (1.13, 1.93); 0.005	1.48 (1.13, 1.93); 0.005	1.49 (1.14, 1.95); 0.004	1.49 (1.14, 1.95); 0.004[a]
Intermediate (N=5274)	156/32 058	2.10 (1.62, 2.72); <0.001	2.10 (1.62, 2.72); <0.001	2.02 (1.56, 2.62); <0.001	2.03 (1.56, 2.63); <0.001[a]
Low (N=5114)	179/30 727	2.21 (1.72, 2.85); <0.001	2.19 (1.70, 2.82); <0.001	1.95 (1.51, 2.52); <0.001	1.96 (1.52, 2.53); <0.001[a]
**High genetic risk**					
High (N=6076)	216/36 961	2.53 (1.98, 3.24); <0.001	2.54 (1.98, 3.24); <0.001	2.55 (1.99, 3.26); <0.001	2.55 (1.99, 3.26); <0.001[a]
Intermediate (N=5056)	219/30 489	3.10 (2.42, 3.96); <0.001	3.10 (2.42, 3.96); <0.001	3.02 (2.37, 3.87); <0.001	3.03 (2.37, 3.87); <0.001[a]
Low (N=4981)	304/29 489	4.09 (3.23, 5.17); <0.001	4.07 (3.21, 5.15); <0.001	3.66 (2.89, 4.64); <0.001	3.68 (2.90, 4.66); <0.001[a]
P value for trend		*P <0.001*	*P <0.001*	*P <0.001*	*P <0.001*

Abbreviation: AF, atrial fibrillation; BMI, body mass index; CI, confidence interval; FDR, false-discovery rate; HR, hazard ratio.

Analyses were conducted using Cox proportional hazard models. **Model 1** was adjusted for age, sex, and first 10 principal components of ancestry. **Model 2** was adjusted as in model 1 and for Townsend deprivation index, recruitment centre, education level, and season of accelerometer wear. **Model 3** was adjusted as in model 2 and for BMI categories, healthy diet score, smoking status, alcohol intake, coffee consumption, tea consumption, hypertension, diabetes, and dyslipidemia. **Model 4** was adjusted as in model 3 and for sleep efficiency and sleep duration. Participants with prevalent AF were excluded. [a] P values remained significant after multiple testing with FDR method.

Supplementary Table 10. Risk of Incident atrial fibrillation according to circadian rest-activity characteristics within each genetic risk category

Subgroup	No. of AF cases/ person-years	Model 1 HR (95% CI); *P*	Model 2 HR (95% CI); *P*	Model 3 HR (95% CI); *P*	Model 4 HR (95% CI); *P*	P for interaction
**Amplitude**						0.76
**Low genetic risk**						
High (N=7121)	89/43 571	1[Reference]	1[Reference]	1[Reference]	1[Reference]	
Intermediate (N=7673)	134/46 802	1.34 (1.03, 1.76); 0.03	1.35 (1.03, 1.77); 0.03	1.31 (1.00, 1.71); 0.05	1.30 (1.00, 1.71); 0.05	
Low (N=6480)	187/38 993	1.76 (1.36, 2.26); <0.001	1.76 (1.36, 2.27); <0.001	1.54 (1.19, 2.00); 0.001	1.52 (1.17, 1.97); 0.002[a]	
P value for trend		*P <0.001*	*P <0.001*	*P =0.001*	*P =0.002*	
**Intermediate genetic risk**						
High (N=6976)	134/42 643	1[Reference]	1[Reference]	1[Reference]	1[Reference]	
Intermediate (N=7667)	183/46 750	1.20 (0.96, 1.50); 0.12	1.20 (0.96, 1.50); 0.12	1.16 (0.93, 1.46); 0.18	1.17 (0.94, 1.47); 0.17	
Low (N=6448)	244/38 578	1.57 (1.26, 1.94); <0.001	1.57 (1.26, 1.94); <0.001	1.39 (1.12, 1.73); 0.003	1.38 (1.11, 1.72); 0.004[a]	
P value for trend		*P <0.001*	*P <0.001*	*P =0.003*	*P =0.004*	
**High genetic risk**						
High (N=7040)	243/42 780	1[Reference]	1[Reference]	1[Reference]	1[Reference]	
Intermediate (N=7316)	313/44 078	1.26 (1.06, 1.49); 0.008	1.26 (1.06, 1.49); 0.008	1.23 (1.04, 1.45); 0.02	1.23 (1.04, 1.45); 0.02[a]	
Low (N=6206)	393/36 816	1.56 (1.33, 1.83); <0.001	1.55 (1.32, 1.82); <0.001	1.39 (1.18, 1.64); <0.001	1.39 (1.18, 1.64); <0.001[a]	
P value for trend		*P <0.001*	*P <0.001*	*P <0.001*	*P <0.001*	
**Acrophase**						0.30
**Low genetic risk**						
Advanced (N=4386)	109/26 489	1[Reference]	1[Reference]	1[Reference]	1[Reference]	
Intermediate (N=6830)	133/41 374	1.01 (0.78, 1.30); 0.97	1.00 (0.78, 1.30); 0.97	1.02 (0.79, 1.32); 0.87	1.03 (0.80, 1.33); 0.82	

Delayed (N=10 058)	168/61 503	1.01 (0.79, 1.29); 0.92	1.01 (0.79, 1.30); 0.91	1.02 (0.80, 1.31); 0.87	1.03 (0.81, 1.32); 0.79	
P value for trend		P=0.92	P=0.91	P=0.88	P=0.80	
**Intermediate genetic risk**						
Advanced (N=4291)	113/25 949	1[Reference]	1[Reference]	1[Reference]	1[Reference]	
Intermediate (N=6613)	180/40 071	1.28 (1.01, 1.62); 0.04	1.29 (1.02, 1.63); 0.04	1.30 (1.02, 1.64); 0.03	1.31 (1.03, 1.66); 0.03[a]	
Delayed (N=10 187)	268/61 950	1.41 (1.13, 1.76); 0.002	1.43 (1.14, 1.79); 0.002	1.41 (1.13, 1.76); 0.003	1.43 (1.14, 1.79); 0.002[a]	
P value for trend		P=0.003	P=0.002	P=0.004	P=0.003	
**High genetic risk**						
Advanced (N=4066)	199/24 289	1[Reference]	1[Reference]	1[Reference]	1[Reference]	
Intermediate (N=6583)	322/39 582	1.24 (1.04, 1.48); 0.02	1.27 (1.06, 1.52); 0.009	1.28 (1.07, 1.53); 0.007	1.29 (1.08, 1.54); 0.006[a]	
Delayed (N=9913)	428/59 802	1.22 (1.03, 1.45); 0.02	1.24 (1.05, 1.47); 0.01	1.23 (1.04, 1.46); 0.02	1.24 (1.04, 1.47); 0.02[a]	
P value for trend		P=0.04	P=0.03	P=0.04	P=0.04	
**F-Pseudo**						0.80
**Low genetic risk**						
High (N=5026)	96/30 676	1[Reference]	1[Reference]	1[Reference]	1[Reference]	
Intermediate (N=7092)	141/43 111	1.00 (0.77, 1.29); 0.97	0.99 (0.76, 1.29); 0.95	0.99 (0.76, 1.29); 0.95	0.99 (0.76, 1.29); 0.94	
Low (N=9156)	173/55 579	1.03 (0.80, 1.32); 0.84	1.03 (0.80, 1.32); 0.84	1.01 (0.78, 1.29); 0.97	1.00 (0.77, 1.28); 0.98	
P value for trend		P=0.81	P=0.82	P=0.96	P=0.99	
**Intermediate genetic risk**						
High (N=5082)	136/30 905	1[Reference]	1[Reference]	1[Reference]	1[Reference]	
Intermediate (N=6959)	181/42 269	0.94 (0.76, 1.18); 0.61	0.94 (0.75, 1.17); 0.56	0.92 (0.74, 1.15); 0.46	0.92 (0.74, 1.15); 0.46	
Low (N=9050)	244/54 797	1.01 (0.82, 1.25); 0.92	1.00 (0.81, 1.24); 0.98	0.97 (0.78, 1.20); 0.76	0.96 (0.78, 1.19); 0.70	
P value for trend		P=0.83	P=0.88	P=0.86	P=0.79	
**High genetic risk**						

High (N=4899)	207/29 725	1[Reference]	1[Reference]	1[Reference]	1[Reference]	
Intermediate (N=6833)	323/40 972	1.05 (0.88, 1.25); 0.56	1.07 (0.90, 1.28); 0.45	1.04 (0.88, 1.24); 0.64	1.04 (0.88, 1.24); 0.64	
Low (N=8830)	419/52 976	1.10 (0.93, 1.30); 0.28	1.10 (0.93, 1.30); 0.28	1.06 (0.89, 1.25); 0.52	1.05 (0.89, 1.25); 0.54	
P value for trend		P=0.28	P=0.30	P=0.53	P=0.56	
**Mesor**						0.47
**Low genetic risk**						
High (N=8028)	112/49 093	1[Reference]	1[Reference]	1[Reference]	1[Reference]	
Intermediate (N=6664)	125/40 575	1.34 (1.03, 1.73); 0.03	1.35 (1.04, 1.74); 0.02	1.30 (1.00, 1.68); 0.047	1.30 (1.00, 1.68); 0.047[a]	
Low (N=6582)	173/39 698	1.66 (1.31, 2.11); <0.001	1.66 (1.31, 2.11); <0.001	1.46 (1.14, 1.86); 0.002	1.45 (1.14, 1.86); 0.003[a]	
P value for trend		*P <0.001*	*P <0.001*	P=0.003	P=0.003	
**Intermediate genetic risk**						
High (N=7875)	156/48 094	1[Reference]	1[Reference]	1[Reference]	1[Reference]	
Intermediate (N=6707)	189/40 769	1.44 (1.16, 1.78); <0.001	1.44 (1.16, 1.78); <0.001	1.38 (1.11, 1.70); 0.003	1.38 (1.12, 1.71); 0.003[a]	
Low (N=6509)	216/39 107	1.50 (1.22, 1.84); <0.001	1.50 (1.22, 1.84); <0.001	1.33 (1.07, 1.64); 0.009	1.33 (1.07, 1.64); 0.009[a]	
P value for trend		*P <0.001*	*P <0.001*	P=0.012	P=0.011	
**High genetic risk**						
High (N=7852)	290/47 649	1[Reference]	1[Reference]	1[Reference]	1[Reference]	
Intermediate (N=6385)	287/38 474	1.23 (1.05, 1.45); 0.01	1.23 (1.04, 1.45); 0.01	1.19 (1.01, 1.40); 0.04	1.19 (1.01, 1.40); 0.04[a]	
Low (N=6325)	372/37 551	1.49 (1.28, 1.74); <0.001	1.48 (1.27, 1.73); <0.001	1.33 (1.13, 1.55); <0.001	1.33 (1.14, 1.56); <0.001[a]	
P value for trend		*P <0.001*	*P <0.001*	*P <0.001*	*P <0.001*	

Abbreviation: AF, atrial fibrillation; BMI, body mass index; CI, confidence interval; FDR, false-discovery rate; HR, hazard ratio.

Analyses were conducted using Cox proportional hazard models. **Model 1** was adjusted for age, sex, and first 10 principal components of ancestry. **Model 2** was adjusted as in model 1 and for Townsend deprivation index, recruitment centre, education level, and season of accelerometer wear. **Model 3** was adjusted as in model 2 and for BMI categories, healthy diet score, smoking status, alcohol intake, coffee consumption, tea consumption, hypertension, diabetes, and dyslipidemia. **Model 4** was adjusted as in model 3 and for sleep efficiency and sleep duration. Participants with prevalent AF were excluded. [a] P values remained significant after multiple testing with FDR method.

Supplementary Table 11. Single-nucleotide polymorphisms used to build the genetic risk score for atrial fibrillation

Rs ID	Position	Risk/reference allele	Effect (beta)	StdErr	P-value
rs284277	chr1:10790797	C/A	0.0422	0.0069	1.245e-09
rs7529220	chr1:22282619	C/T	0.0621	0.0098	1.983e-10
rs2885697	chr1:41544279	G/T	0.0439	0.007	2.884e-10
rs11590635	chr1:49309764	A/G	0.1456	0.0248	4.123e-09
rs146518726	chr1:51535039	A/G	0.1605	0.0207	8.27e-15
rs1545300	chr1:112464004	C/T	0.0558	0.0073	1.481e-14
rs4073778	chr1:116297758	A/C	0.0486	0.0067	4.956e-13
rs10465885	chr1:147232740	C/T	0.0302	0.0067	5.735e-06
rs79187193	chr1:147255831	G/A	0.1162	0.0153	3.15e-14
rs6689306	chr1:154395946	A/G	0.046	0.0068	1.36e-11
rs4999127	chr1:154714006	A/G	0.0827	0.0098	4.279e-17
rs11264280	chr1:154862952	T/C	0.1347	0.0071	3.067e-79
rs72700114	chr1:170193825	C/G	0.2021	0.013	3.288e-54
rs72700118	chr1:170194823	A/C	0.1227	0.0101	9.524e-34
rs577676	chr1:170587340	C/T	0.0923	0.0067	1.62e-43
rs10753933	chr1:203026214	T/G	0.0609	0.0067	9.844e-20
rs4951258	chr1:205691316	A/G	0.0376	0.0067	2.104e-08
rs7578393	chr2:26165528	T/C	0.0614	0.0088	2.42e-12
rs11689011	chr2:46541176	T/C	0.0321	0.0103	3.124e-08
rs11125871	chr2:61470126	C/T	0.0394	0.0068	6.416e-09
rs2540949	chr2:65284231	A/T	0.0659	0.0068	2.949e-22
rs6747542	chr2:70106832	T/C	0.0554	0.0067	1.096e-16
rs72926475	chr2:86594487	G/A	0.0683	0.0102	2.372e-11
rs28387148	chr2:127433465	T/C	0.0741	0.0113	6.251e-11
rs67969609	chr2:145760353	G/C	0.0711	0.0126	1.712e-08
rs56181519	chr2:175555714	C/T	0.0662	0.0077	6.463e-18
rs2288327	chr2:179411665	G/A	0.0919	0.0089	7.257e-25
rs3820888	chr2:201180023	C/T	0.0684	0.0068	5.748e-24
rs35544454	chr2:213266003	A/T	0.0589	0.0087	1.1e-11
rs7650482	chr3:12841804	G/A	0.0711	0.007	1.793e-24
rs73041705	chr3:24463235	T/C	0.0443	0.0073	1.546e-09
rs7374540	chr3:38634142	A/C	0.0325	0.0068	1.682e-06
rs7373065	chr3:38710315	T/C	0.2024	0.0251	7.584e-16
rs6790396	chr3:38771925	G/C	0.0627	0.0068	2.401e-20
rs34080181	chr3:66454191	G/A	0.0446	0.0069	1.278e-10
rs17005647	chr3:69406181	T/C	0.0413	0.0069	2.698e-09
rs6771054	chr3:89489529	T/C	0.0457	0.0068	2.424e-11
rs10804493	chr3:111554426	A/G	0.0558	0.007	1.629e-15
rs1278493	chr3:135814009	G/A	0.0389	0.0068	8.769e-09

rs13077048	chr3:141106954	T/A	0.0493	0.0104	4.75e-10
rs62274627	chr3:148702947	A/G	0.0297	0.0109	2.458e-08
rs7612445	chr3:179172979	T/G	0.0493	0.0084	4.808e-09
rs60902112	chr3:194800853	T/C	0.0445	0.0079	1.719e-08
rs34104130	chr4:10101300	G/T	0.0365	0.0112	2.552e-11
rs1458038	chr4:81164723	T/C	0.0434	0.0072	1.735e-09
rs6841049	chr4:83910712	T/G	0.0242	0.0103	1.954e-08
rs10006327	chr4:103890980	C/T	0.0364	0.0067	4.423e-08
rs61501369	chr4:111524629	T/C	0.1038	0.008	4.686e-38
rs6850025	chr4:111596360	A/G	0.1815	0.0154	4.93e-32
rs67249485	chr4:111699685	T/A	0.3655	0.0081	7.32e-443
rs3853445	chr4:111761487	T/C	0.1693	0.0076	3.60e-109
rs79399769	chr4:111925656	C/T	0.1197	0.0232	2.591e-07
rs1532170	chr4:112165212	G/A	0.0306	0.007	1.258e-05
rs138311480	chr4:112454295	C/T	0.0739	0.0284	0.00928
rs114904067	chr4:112604821	G/A	0.082	0.0215	0.0001364
rs7687819	chr4:113329345	A/G	0.0218	0.0079	0.00612
rs6829664	chr4:114448656	G/A	0.0556	0.0076	1.922e-13
rs10213171	chr4:148937537	G/C	0.091	0.0134	1.323e-11
rs10520260	chr4:174447349	A/G	0.0457	0.0073	3.362e-10
rs12648245	chr4:174641184	T/C	0.0926	0.0127	3.454e-13
rs6596717	chr5:106427609	C/A	0.0404	0.0068	3e-09
rs337705	chr5:113737062	G/T	0.0564	0.0068	1.634e-16
rs2012809	chr5:128190363	G/A	0.0582	0.0094	4.923e-10
rs2040862	chr5:137419989	T/C	0.1084	0.0087	1.077e-35
rs17118812	chr5:139703286	C/T	0.0431	0.0114	1.827e-10
rs6580277	chr5:142818123	G/A	0.067	0.0079	1.643e-17
rs12188351	chr5:168386089	A/G	0.0865	0.0145	2.516e-09
rs6891790	chr5:172670745	G/T	0.0729	0.0076	4.531e-22
rs28439930	chr5:173393111	G/C	0.0458	0.0068	1.186e-11
rs73366713	chr6:16415751	G/A	0.1035	0.0099	1.531e-25
rs2308655	chr6:31322303	C/G	0.0494	0.012	8.085e-09
rs3176326	chr6:36647289	G/A	0.0626	0.0085	1.424e-13
rs12211255	chr6:76188330	A/C	0.057	0.0159	2.596e-10
rs2031522	chr6:87821501	A/G	0.0436	0.0068	1.465e-10
rs3951016	chr6:118559658	A/T	0.0648	0.0067	2.149e-22
rs9401451	chr6:122099152	G/A	0.0733	0.011	2.513e-11
rs13195459	chr6:122403559	G/A	0.0623	0.007	4.152e-19
rs4896104	chr6:135119089	C/T	0.0421	0.0103	8.615e-10
rs117984853	chr6:149399100	T/G	0.1228	0.012	1.342e-24
rs12700233	chr7:904757	T/G	0.0367	0.0104	5.204e-11
rs55734480	chr7:14372009	A/G	0.0548	0.0078	2.201e-12
rs6462079	chr7:28415827	A/G	0.0466	0.0076	8.793e-10

rs35005436	chr7:74134911	C/T	0.0612	0.0097	3.342e-10
rs56201652	chr7:92278116	G/A	0.0531	0.0075	1.743e-12
rs2283038	chr7:106835410	T/C	0.0266	0.012	9.756e-10
rs11773845	chr7:116191301	A/C	0.1054	0.0067	2.39e-55
rs55985730	chr7:128417044	G/T	0.0867	0.0149	5.236e-09
rs7789146	chr7:150661409	G/A	0.0584	0.0087	2.116e-11
rs35620480	chr8:11499908	C/A	0.054	0.0092	5.152e-09
rs7508	chr8:17913970	A/G	0.0711	0.0075	1.691e-21
rs7834729	chr8:21821778	G/T	0.0653	0.0104	3.553e-10
rs17430364	chr8:118863445	T/A	0.0425	0.0133	4.433e-08
rs62521286	chr8:124551975	G/A	0.1202	0.0135	4.498e-19
rs4871397	chr8:124635197	G/C	0.0756	0.0138	4.646e-08
rs35006907	chr8:125859817	A/C	0.0083	0.0107	2.663e-08
rs72721963	chr8:135798224	G/A	0.039	0.0174	1.907e-09
rs6994744	chr8:141740868	C/A	0.0405	0.0066	1.095e-09
rs10821415	chr9:97713459	A/C	0.0821	0.0067	2.924e-34
rs4743034	chr9:109632353	A/G	0.0246	0.0122	6.139e-09
rs10760361	chr9:127178266	G/T	0.0259	0.0106	6.035e-10
rs2274115	chr9:139094773	G/A	0.0487	0.0076	1.691e-10
rs12245149	chr10:65321147	C/A	0.047	0.0067	1.664e-12
rs7096385	chr10:69664881	T/C	0.0707	0.013	4.871e-08
rs60212594	chr10:75414344	G/C	0.1176	0.0096	9.199e-35
rs10458660	chr10:77936576	G/A	0.0537	0.0087	6.783e-10
rs55693294	chr10:105277474	T/C	0.0546	0.0146	0.0001819
rs11598047	chr10:105342672	G/A	0.1537	0.009	8.952e-66
rs35176054	chr10:105480387	A/T	0.1391	0.01	3.207e-44
rs10749053	chr10:112576695	T/C	0.0555	0.0097	1.049e-08
rs10741807	chr11:20011445	T/C	0.0729	0.0079	1.591e-20
rs565449	chr11:95092398	G/A	0.0409	0.0109	2.235e-09
rs4935786	chr11:121661507	T/A	0.0463	0.0079	4.854e-09
rs76097649	chr11:128764570	A/G	0.1151	0.0124	1.26e-20
rs2291437	chr12:24715048	G/T	0.0955	0.0104	5.052e-20
rs4963776	chr12:24779491	G/T	0.0913	0.0088	1.839e-25
rs17380837	chr12:26345526	C/T	0.0501	0.0072	4.799e-12
rs12809354	chr12:32978437	C/T	0.0718	0.0094	2.886e-14
rs11614818	chr12:56055815	C/T	0.0329	0.007	2.441e-06
rs2860482	chr12:57105938	A/C	0.054	0.0076	1.212e-12
rs71454237	chr12:70013415	G/A	0.062	0.0084	1.784e-13
rs775498	chr12:70071513	G/A	0.0423	0.0074	1.053e-08
rs12426679	chr12:76237987	C/T	0.0391	0.0067	4.945e-09
rs883079	chr12:114793240	T/C	0.0981	0.0074	2.837e-40
rs116904997	chr12:120668534	G/A	0.1116	0.0405	1.275e-08
rs10773657	chr12:123327900	C/A	0.0575	0.0103	2.537e-08

rs7134121	chr12:124447346	T/C	0.0317	0.0108	2.003e-11
rs6560886	chr12:133150210	C/T	0.051	0.009	1.491e-08
rs9506925	chr13:23368943	T/C	0.0449	0.0075	2.716e-09
rs1980728	chr13:47247985	G/T	0.0361	0.0116	2.083e-09
rs35569628	chr13:113872712	T/C	0.0452	0.008	1.378e-08
rs422068	chr14:23864804	C/T	0.0439	0.007	3.873e-10
rs1957021	chr14:32924505	C/T	0.0583	0.008	2.274e-13
rs11156751	chr14:32990437	C/T	0.0719	0.0077	6.941e-21
rs73241997	chr14:35173775	T/C	0.0733	0.0093	2.942e-15
rs2738413	chr14:64679960	A/G	0.0778	0.0067	2.546e-31
rs74884082	chr14:73249419	C/T	0.0493	0.0078	3.479e-10
rs10873298	chr14:77426525	C/T	0.0401	0.0069	7.072e-09
rs7170477	chr15:64103777	A/G	0.0393	0.0072	4.977e-08
rs745636	chr15:70457720	G/A	0.0392	0.0122	7.159e-10
rs74022964	chr15:73677264	T/C	0.1132	0.009	3.51e-36
rs12908004	chr15:80676925	G/A	0.0732	0.009	4.115e-16
rs2759301	chr15:80994288	A/G	0.039	0.0067	5.038e-09
rs4965430	chr15:99268850	C/G	0.0441	0.0069	1.258e-10
rs118159104	chr16:1676804	G/T	0.1737	0.0325	9.275e-08
rs140185678	chr16:2003016	A/G	0.1659	0.0218	2.434e-14
rs77316573	chr16:2265271	T/C	0.0529	0.0089	3.265e-09
rs2359171	chr16:73053022	A/T	0.1746	0.0086	4.65e-91
rs876727	chr16:73067761	T/G	0.084	0.0084	1.974e-23
rs7225165	chr17:1309850	G/A	0.0655	0.0111	3.203e-09
rs9899183	chr17:7452977	T/C	0.0452	0.0075	2.017e-09
rs72811294	chr17:12618680	G/C	0.072	0.0106	9.67e-12
rs11658278	chr17:38031164	T/C	0.0443	0.0067	3.465e-11
rs12604076	chr17:76773638	T/C	0.0365	0.0066	3.627e-08
rs9953366	chr18:46474192	C/T	0.049	0.0073	1.823e-11
rs9963878	chr18:48679522	C/T	0.0653	0.012	4.845e-08
rs8088085	chr18:48708548	A/C	0.0365	0.0067	4.794e-08
rs2974231	chr19:48170757	A/G	0.033	0.0104	1.107e-09
rs2145274	chr20:6572014	A/C	0.0169	0.0231	7.469e-12
rs2834618	chr21:36119111	T/G	0.0944	0.0112	3.412e-17
rs56040242	chr21:45766944	A/G	0.0417	0.0121	1.264e-08
rs464901	chr22:18597502	T/C	0.0508	0.0072	1.531e-12
rs133902	chr22:26164079	T/C	0.0419	0.0068	9.137e-10
Taken from: Nielsen JB, et al. Biobank-driven genomic discovery yields new insight into atrial fibrillation biology. Nat Genet. 2018;50(9):1234-1239.					

Supplementary Table 12. Codes used in the UK Biobank study to identify atrial fibrillation cases

Data fields	Field names	Data codes	Data code definitions
20002	Non-cancer illness code, self-report	1471, 1483	Atrial fibrillation, Atrial flutter
41271	Diagnoses - ICD9	4273	Atrial fibrillation and flutter
41270, 40001, 40002	Diagnoses - ICD10 Primary cause of death: ICD10, Secondary cause of death: ICD10	I48, I48.0, I48.1, I48.2, I48.3, I48.4, I48.9	Atrial fibrillation and flutter, Paroxysmal atrial fibrillation, Persistent atrial fibrillation, Chronic atrial fibrillation, Typical atrial flutter, Atypical atrial flutter, Atrial fibrillation and atrial flutter, unspecified
41272	Operative procedures	K62.1, K62.2, K62.3, K62.4	Percutaneous transluminal ablation of pulmonary vein to left atrium conducting system, Percutaneous transluminal ablation of atrial wall for atrial flutter, Percutaneous transluminal ablation of conducting system of heart for atrial flutter NEC, Percutaneous transluminal internal cardioversion NEC

Supplementary Table 13. The numbers (percentages) of participants with missing covariates

Covariates	N	%
Any missing covariate	721	1.15
Age at accelerometry (years)	0	0.00
Female	0	0.00
Townsend deprivation index	73	0.12
Recruitment regions	0	0.00
Education level	178	0.28
Season of accelerometer wear	0	0.00
Body mass index categories	89	0.14
Healthy diet score	1	0.002
Smoking status	108	0.17
Alcohol consumption	14	0.02
Coffee consumption	0	0.00
Tea consumption	37	0.06
Hypertension history	39	0.06
Diabetes history	51	0.08
Dyslipidemia history	0	0.00
Sleep efficiency (%)	150	0.24
Sleep duration	150	0.24
