# Supplementary material for: Association of accelerometer-derived circadian abnormalities and genetic risk with incidence of atrial fibrillation
Source: NPJ Digit Med. 2023 Mar 4;6:31. doi: 10.1038/s41746-023-00781-3 (PMC9984286; doi:10.1038/s41746-023-00781-3)
Supplement: Supplementary file 2 — REPORTING SUMMARY [file 41746_2023_781_MOESM2_ESM.pdf]

nature portfolio

Corresponding author(s): Jihui Zhang

Last updated by author(s): Feb 13, 2023

Reporting Summary

Nature Portfolio wishes to improve the reproducibility of the work that we publish. This form provides structure for consistency and transparency in reporting. For further information on Nature Portfolio policies, see our Editorial Policies and the Editorial Policy Checklist.

Statistics

For all statistical analyses, confirm that the following items are present in the figure legend, table legend, main text, or Methods section.

n/a	Confirmed	
☐	☒	The exact sample size (*n*) for each experimental group/condition, given as a discrete number and unit of measurement
☐	☒	A statement on whether measurements were taken from distinct samples or whether the same sample was measured repeatedly
☐	☒	The statistical test(s) used AND whether they are one- or two-sided<br>*Only common tests should be described solely by name; describe more complex techniques in the Methods section.*
☐	☒	A description of all covariates tested
☐	☒	A description of any assumptions or corrections, such as tests of normality and adjustment for multiple comparisons
☐	☒	A full description of the statistical parameters including central tendency (e.g. means) or other basic estimates (e.g. regression coefficient) AND variation (e.g. standard deviation) or associated estimates of uncertainty (e.g. confidence intervals)
☐	☒	For null hypothesis testing, the test statistic (e.g. *F*, *t*, *r*) with confidence intervals, effect sizes, degrees of freedom and *P* value noted<br>*Give P values as exact values whenever suitable.*
☒	☐	For Bayesian analysis, information on the choice of priors and Markov chain Monte Carlo settings
☒	☐	For hierarchical and complex designs, identification of the appropriate level for tests and full reporting of outcomes
☐	☒	Estimates of effect sizes (e.g. Cohen's *d*, Pearson's *r*), indicating how they were calculated

Our web collection on statistics for biologists contains articles on many of the points above.

Software and code

Policy information about availability of computer code

Data collection	All data in this study were derived from the UK Biobank.
Data analysis	R version 4.0.4; SPSS version 26; R studio version 1.4.1717

For manuscripts utilizing custom algorithms or software that are central to the research but not yet described in published literature, software must be made available to editors and reviewers. We strongly encourage code deposition in a community repository (e.g. GitHub). See the Nature Portfolio guidelines for submitting code & software for further information.

Data

Policy information about availability of data

All manuscripts must include a data availability statement. This statement should provide the following information, where applicable:

- Accession codes, unique identifiers, or web links for publicly available datasets
- A description of any restrictions on data availability
- For clinical datasets or third party data, please ensure that the statement adheres to our policy

The UK Biobank data are available from the UK Biobank can be accessed by researchers on application (www.ukbiobank.ac.uk/).

Human research participants

Policy information about studies involving human research participants and Sex and Gender in Research.

Reporting on sex and gender	The sex information is acquired from central registry at recruitment, but in some cases updated by the participant. Hence it may contain a mixture of the sex the National Health Service (NHS) had recorded for the participant and self-reported sex. Accelerometer-measured circadian rest-activity rhythm abnormalities, characterized by decreased strength and height, and later timing of peak activity of circadian rhythm were associated with a higher risk of AF in general population. We also conducted subgroup analyses on sex and found that the findings apply to males and females.
Population characteristics	A total of 62 927 participants were included in our study. The mean (SD) age was 62.48 (7.75) years, and 35 323 (56.13%) were female. During a median (interquartile range) follow-up of 6.16 (5.60-6.68) years, 1920 participants (3.05%) developed atrial fibrillation. Compared with participants without incident atrial fibrillation, those with incident atrial fibrillation were more likely to be older, male, more materially deprived, and English. They also tended to be more obese, smokers, have lower education levels, have a less healthy diet, have more coffee and tea, and were more likely to have hypertension, diabetes, and dyslipidaemia. Regarding the alcohol consumption, participants with incident atrial fibrillation more rarely consumed alcohol two or fewer times per week but more often reported drinking alcohol at least three times per week compared to those without incident atrial fibrillation. In addition, participants with incident atrial fibrillation appeared to have lower sleep efficiency and abnormal sleep duration (<7 hours/day or >8 hours/day).
Recruitment	The UK Biobank is a population-based prospective study with over 500 000 participants aged 40-73 years recruited in 2006-2010. Participants underwent detailed baseline assessments including various socio-demographics, lifestyles, health, and physical assessments through touch-screen questionnaires and physical measurements. Further details of the study are available online (www.ukbiobank.ac.uk). Between February 2013 and December 2015 (on average, approximately 5.5 years after their baseline recruitment), 236 519 UK Biobank participants were invited to participate in an accelerometer study. A total of 106 053 (44.8%) participants agreed to take part and were provided with a wrist-worn Axivity AX3 accelerometer (Axivity, Newcastle upon Tyne, UK). Finally, 103 712 raw accelerometer datasets were received for data analysis.
Ethics oversight	UK Biobank received ethical approval from the NHS (National Health Service) National Research Ethics Service (Ref11/NW/0382). All participants gave written informed consent before enrolment in the study, which was conducted in accord with the principles of the Declaration of Helsinki.

Note that full information on the approval of the study protocol must also be provided in the manuscript.

Field-specific reporting

Please select the one below that is the best fit for your research. If you are not sure, read the appropriate sections before making your selection.

☒ Life sciences ☐ Behavioural & social sciences ☐ Ecological, evolutionary & environmental sciences

For a reference copy of the document with all sections, see nature.com/documents/nr-reporting-summary-flat.pdf

Life sciences study design

All studies must disclose on these points even when the disclosure is negative.

Sample size	Our final sample consisted of N=62 927participants with accelerometer and genotype data from the UK Biobank.
Data exclusions	Based on the data quality metrics provided by the UK Biobank accelerometer working group, the exclusion criteria are as follows: 1) those data flagged by UK Biobank as being unreliable due to unexpectedly small or large size; 2) those with accelerometer data for less than 72 h or did not provide data for all 1-h periods within a 24-h cycle during the 7-day data collection; 3) those data identified by UK Biobank as not well-calibrated; 4) those data were recalibrated using the previous accelerometer record from the same device worn by a different participant; 5) those data with a non-zero count of interrupted recording periods; 6) those data with more than 768 (Q3 + 1.5×IQR) data recording errors. Furthermore, during the quality control process of genetic data, participants with missingness (>10%), outliers for heterozygosity, biologically related, and those whose reported sex was inconsistent with sex inferred from the genetic data as well as those with sex chromosome aneuploidy, and those who were genetically defined as not white British, and those with prevalent atrial fibrillation based on self-report or medical records were also excluded. Finally, 62 927 participants were included in our study.
Replication	This is a population-based cohort study, and we have not yet replicated the findings in other samples. However, we used different statistical methods to verify our findings: 1) controlling for a wide range of potential covariates; 2) comprehensive sensitivity analyses (e.g., using competing risk regression models, excluding events within the first year of follow-up); 3) validated in multiple testing correction by the Benjamini-Hochberg False Discovery Rate method. Our main findings were quite robust and consistent across these analyses.
Randomization	This is an observational cohort study, and randomization is not applicable in this study.
Blinding	Given that this is a non-experimental study, blinding was not used or necessary.

Reporting for specific materials, systems and methods

We require information from authors about some types of materials, experimental systems and methods used in many studies. Here, indicate whether each material, system or method listed is relevant to your study. If you are not sure if a list item applies to your research, read the appropriate section before selecting a response.

Materials & experimental systems

n/a	Involved in the study
☒	☐ Antibodies
☒	☐ Eukaryotic cell lines
☒	☐ Palaeontology and archaeology
☒	☐ Animals and other organisms
☒	☐ Clinical data
☒	☐ Dual use research of concern

Methods

n/a	Involved in the study
☒	☐ ChIP-seq
☒	☐ Flow cytometry
☒	☐ MRI-based neuroimaging
